# A chimeric protein-based malaria vaccine candidate induces robust T cell responses against *Plasmodium vivax* MSP1_19_

**DOI:** 10.1038/srep34527

**Published:** 2016-10-06

**Authors:** Jairo Andres Fonseca, Monica Cabrera-Mora, Balwan Singh, Joseli Oliveira-Ferreira, Josué da Costa Lima-Junior, J. Mauricio Calvo-Calle, Jose Manuel Lozano, Alberto Moreno

**Affiliations:** 1 Emory Vaccine Center, YerkesNational Primate Research Center, Emory University, 954 Gatewood Road, Atlanta, GA 30329, USA; 2 Division of Infectious Diseases, Department of Medicine, Emory University, 69 Jesse Hill, Jr. Drive, SE, Atlanta, GA 30303, USA; 3 Laboratory of Immunoparasitology, Oswaldo Cruz Institute, Oswaldo Cruz Foundation, (FIOCRUZ), Rio de Janeiro, RJ, Brazil; 4 Department of Pathology, University of Massachusetts Medical School, Worcester, MA, USA; 5 Molecular Mimetism of Infectious Agents Unit, Department of Pharmacy, Universidad Nacional de Colombia, Bogota, Colombia.

## Abstract

The most widespread *Plasmodium* species, *Plasmodium vivax*, poses a significant public health threat. An effective vaccine is needed to reduce global malaria burden. Of the erythrocytic stage vaccine candidates, the 19 kDa fragment of the *P. vivax* Merozoite Surface Protein 1 (PvMSP1_19_) is one of the most promising. Our group has previously defined several promiscuous T helper epitopes within the PvMSP1 protein, with features that allow them to bind multiple MHC class II alleles. We describe here a *P. vivax* recombinant modular chimera based on MSP1 (PvRMC-MSP1) that includes defined T cell epitopes genetically fused to PvMSP1_19_. This vaccine candidate preserved structural elements of the native PvMSP1_19_ and elicited cytophilic antibody responses, and CD4+ and CD8+ T cells capable of recognizing PvMSP1_19_. Although CD8+ T cells that recognize blood stage antigens have been reported to control blood infection, CD8+ T cell responses induced by *P. falciparum* or *P. vivax* vaccine candidates based on MSP1_19_ have not been reported. To our knowledge, this is the first time a protein based subunit vaccine has been able to induce CD8+ T cell against PvMSP1_19_. The PvRMC-MSP1 protein was also recognized by naturally acquired antibodies from individuals living in malaria endemic areas with an antibody profile associated with protection from infection. These features make PvRMC-MSP1 a promising vaccine candidate.

*Plasmodium vivax*, the most widespread species of *Plasmodium*, is responsible for 15 million cases of malaria annually^1^. Most of the funding and resourcing for malaria research have been allocated to *P. falciparum* on the basis that this species is the only human malaria parasite associated with severe disease. Nonetheless, recent reports have shown that *P. vivax* infections are responsible for severe clinical outcomes^2^. Importantly, several strategies used to control *P. falciparum* may not be effective against *P. vivax* malaria since this parasite exhibits unique characteristics that make its transmission more efficacious than that of *P. falciparum*^3^. This effect has been observed in the use of Insecticide Treated Nets (ITN) which exhibit lower efficacy for the prevention of vivax malaria^4^. Additionally, the emergence of *P. vivax* resistance to primaquine^5^ is a major concern in the field as this is the only medication available to cure *P. vivax* infection. The need for an effective *P. vivax* vaccine is, therefore, a public health priority.

The release of new merozoites during the blood stage infection is the main event in the pathophysiology of malaria^6^. Of the *Plasmodium* blood stage antigens studied, MSP1 is one of the best-characterized malaria vaccine candidates. MSP1 is part of a major complex that makes up most of the merozoite surface^7^. The merozoites released from the schizont exhibit a ~200 kDa MSP1-precursor that is cleaved into several fragments that have been characterized in *P. falciparum*. Of these fragments, the C-terminal MSP1-42 kDa fragment remains on the merozoite surface as a dimer attached through a glycosylphosphatidylinositol (GPI) anchor. At the initiation of RBC invasion, the MSP1_42_ kDa fragment is further processed producing two products MSP1_33_ and MSP1_19_, with MSP1_19_ remaining on the parasite surface as a monomer to provide the signal for the parasite to start intracellular development^7^.

Antibodies against MSP1, and in particular to MSP1_19_, can block erythrocyte invasion and replication *in vitro*^8^, protect against parasitemia and malarial anemia^9^,^10^ and lyse merozoites via complement-mediated inhibition^11^. Despite being able to induce of humoral immunity the MSP1_19_ fragment is poorly recognized by T cells^12^,^13^ due to several disulfide bridges which reduce antigen processing and presentation^14^. In contrast, MSP1_33_ specific CD4+ T cells have shown to be involved in protection in the murine model via IFN-γ production, and induction of cytophilic antibodies^15^,^16^. These features have been confirmed in humans as CD4+ T cells able to recognize blood stage antigens have been linked to protection^17^.

Although a cytotoxic response against MSP1 has not been clearly associated with protection, blood stage antigen specific CD8+ T cells are elicited after infection^18^. In the murine model, antigens expressed by *P. berghei* blood stages are processed and cross-presented by CD8α DCs to stimulate CD8+ T cells^19^. In humans, CD8+ T cells induced after vaccination with viral vectors expressing *P. falciparum* MSP1_42_ can prolong the prepatent period, by controlling the parasite in the liver, since liver schizonts express MSP1^20^.

Despite promising results with *P. falciparum* antigens, *P. vivax* blood stage vaccine candidates have not been tested in clinical trials. Preclinical trials in non-human primates have been reported for PvMSP1 based vaccines, showing partial protection with an immunogenicity dependent on the adjuvant used^21^,^22^,^23^. Therefore, more studies are required to obtain a safe, highly immunogenic PvMSP1 formulation.

In previous studies, we defined several CD4+ T cell epitopes within the native PvMSP1 with features of promiscuous T cell epitopes (i.e. epitopes capable of binding to a broad range of MHC class II alleles)^24^. Synthetic peptides representing these T cell epitopes were successfully recognized by lymphocytes from individuals naturally infected with *P. vivax*^24^. To further characterize these epitopes, we designed a *P. yoelii* recombinant modular chimera (PyRMC-MSP1) that included the orthologous sequences of the promiscuous T cell epitopes identified in *P. vivax* which were assembled in tandem and genetically fused to the PyMSP1_19_ protein fragment. Proof-of-concept studies demonstrated that the inclusion of promiscuous T cell epitopes increased the immunogenicity and efficacy against hyperparasitemia and severe anemia induced by two different *P. yoelii* strains^9^.

Based on that evidence, we designed a *P. vivax* recombinant modular chimera based on MSP1 (PvRMC-MSP1) including the five most promiscuous T cell epitopes previously identified using functional assays^24^. These promiscuous T cell epitopes were arrayed in tandem conformation as described for *P. yoelii*^9^ and genetically fused to an elongated PvMSP1_19_ fragment (which includes two predicted T helper epitopes present in MSP1_33_)^9^. PvRMC-MSP1 exhibits a complex tertiary structure while preserving the MSP1_19_ structural features. When administered in a formulation with Montanide ISA 51, the PvRMC-MSP1 protein is able to induce cytophilic antibody responses in the two different mouse strains tested. Importantly, PvRMC-MSP1 can elicit both CD4+ and CD8+ T cells capable of recognizing MSP1_19_. To our knowledge, this is the first time a protein subunit vaccine has been able to induce CD8+ T cells capable of recognizing the 19 kDa fragment of *P. vivax* MSP1.

Furthermore, we observed in seroprevalence studies in a population naturally exposed to malaria, a high frequency of total IgG responders to PvRMC-MSP1 with a predominantly cytophilic IgG1 response. The responses occurred irrespectively of the different HLA haplotypes in the population, suggesting that the PvRMC-MSP1 recognition is not genetically restricted.

In this report, we present the development and immunogenic characteristics of PvRMC-MSP1, a promising *P. vivax* vaccine candidate that merits further development as a component of a multi-stage malaria vaccine.

## Results

### Design, expression and biochemical characterization of PvRMC-MSP1

**Figure 1:**
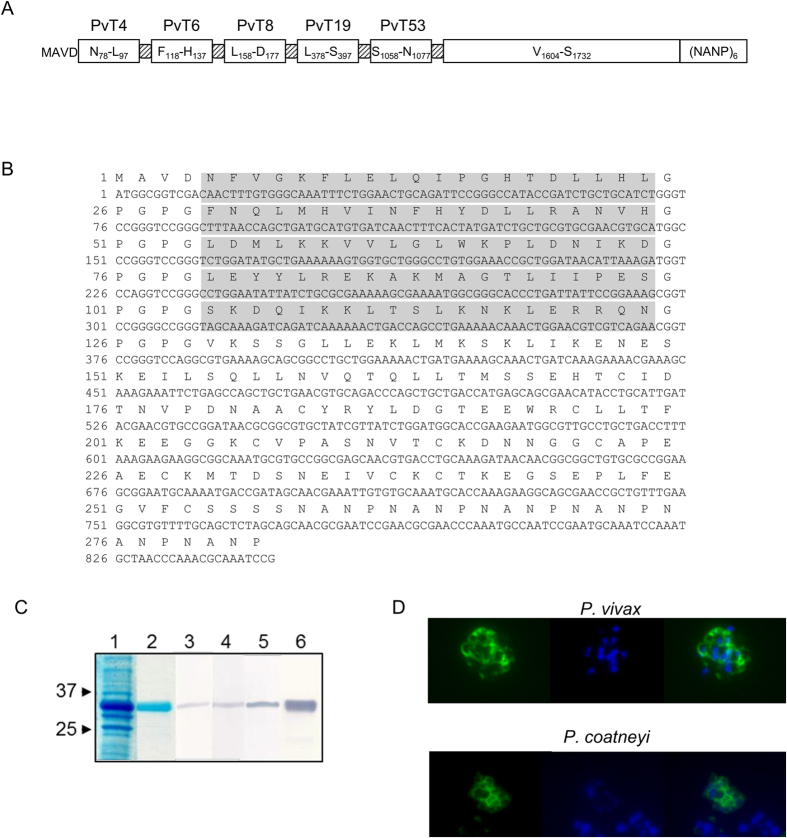
Design, expression and functional characterization of PvRMC-MSP1. (**A**) Schematic representation of the recombinant protein reported here. PvRMC-MSP-1 includes five promiscuous T cell epitopes interspaced with a GPGPG spacers, enclosed in gray boxes, and genetically fused to the carboxyl terminal region of the native sequence that contains EGF domains sequences that are the target of protective antibodies. (**B**) Sequence of the PvRMC-MSP-1 protein. The amino acid sequence is shown in single letter code. The carboxyl terminal (H)6 tag provided by the vector was not included in the sequence. Promiscuous T cell epitopes are highlighted in gray. (**C**) Coomassie stain after SDS-PAGE separation, total bacterial lysate after induction and purified PvRMC-MSP1 are shown separated on a 4–20% gradient gel (lanes 1 and 2). The molecular weight markers (BioRad) are indicated. Western blot analysis of the purified PvRMC-MSP1 (lanes 3–6) incubated with sera samples from mice immunized with a synthetic peptide representing the T cell epitope L_158_-D_177_ (lane 3), a synthetic peptide representing the T cell epitope L_378_-S_397_ (lane 4), a synthetic peptide representing the T cell epitope N_78_-L_97_ (lane 5) or the monoclonal antibody 2A10 that recognizes the C-terminal tag (NANP)_6_ (lane 6). (**D**) Characteristic immunofluorescence pattern of anti-PvRMC-MSP1 IgG antibodies from BALB/c mice at a 1:500 dilution on erythrocytes infected with schizonts from *P. vivax* (Top) or *P. coatneyi* (Bottom). Left panels show anti-PvRMC-MSP1 antibody reactivity using IgG-specific Alexa Fluor 488 goat anti-mouse, center panels show parasite nuclei stained with DAPI. Right panels are a merge of the blue and green fluorescence channels.

The chimeric recombinant PvRMC-MSP1 protein has been developed based on our proof-of-concept studies with *P. yoelii*^9^,^24^,^25^,^26^. The PvMSP1 experimentally defined promiscuous T cell epitopes were arrayed in tandem and genetically fused to an extended PvMSP1_19_ that includes two predicted T helper epitopes present in MSP1_33_ (Fig. 1A,B). We have previously validated the relevance of using this topology for the design of chimeric proteins to improve the immunogenicity of *P. yoelii* and *P. vivax* antigens in murine models^9^,^24^,^25^,^26^,^27^. The promiscuous T cell epitopes (i.e. T helper epitopes able to bind several MHC class II alleles) were experimentally defined in the *P. vivax* Belem strain by peptide binding competition assays and validated using cells collected from naturally infected individuals^24^.

To characterize the biochemical identity of the recombinant PvRMC-MSP1 protein, a panel of polyclonal antibodies generated by immunization with synthetic peptides representing individual T cell epitopes were used for western blot analysis (Fig. 1C). The monoclonal antibody 2A10 which recognizes the *P. falciparum* repeats (NANP)_n_ that were used as a carboxyl terminal tag also recognized the chimeric protein (Fig. 1C, Supplementary Figure S1). The antibodies showed a single band at the expected relative mobility of ~31 kDa. When the SDS-PAGE was performed under reducing conditions, a shift in the mobility was observed for both PvRMC-MSP1 and PvMSP1_19_, indicating that the disulfide bonds present in PvMSP1_19_ that are critical for biological activity are also preserved in PvRMC-MSP1 (Supplementary Figure S2).

In addition to the biochemical assessment, endotoxin levels were also determined using the limulus amebocyte lysate assay. This protein preparation contained 42 endotoxin units (EU)/mg of protein.

### Secondary structure elements on PvRMC-MSP1

Given the relevance of the association between 3D structure and functional activity, we decided to analyze representative secondary structure elements of the PvRMC-MSP1 by circular dichroism (CD). This methodology has proven to be helpful quantitatively to estimate secondary structural elements present in polypeptides, independent of their origin. CD experiments were conducted under different hydrophobic solvent systems, such as water and aqueous 30% TFE (trifluoroethanol), to detect stable structural regions in the entire PvRMC-MSP1 conformation^28^.

**Figure 2:**
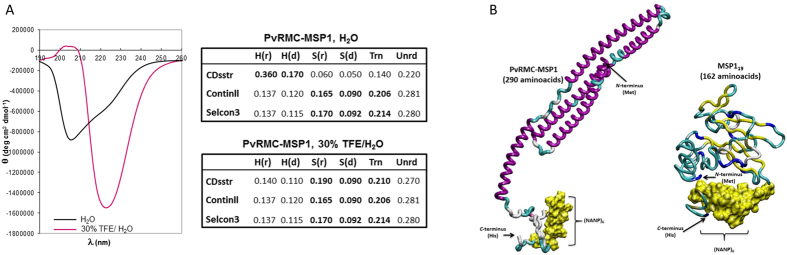
PvRMC-MSP1 Molecular modeling and 3D conformations. (**A**) Secondary structure patterns by circular dichroism and data deconvolution with CDsstr, Continll, and Selcon3 of the CDPro software package. Solid black lane denotes the secondary structure element profile of PvRMC-MSP1 recorded in water, and the solid magenta line indicates the molecule structure recorded in 30% of aqueous TFE. (**B**) PvRMC-MSP1 and PvMSP1_19_ predicted-validated 3D structure molecular model. Purple ribbons represent the polypeptide backbone α-helical conformations, green and white regions represent highly flexible stretches and the yellow and blue zones represent the β-strands and β–turns. In the PvRMC-MSP1 and PvMSP1_19_ structures, the (NANP)_6_ motif is present as the surface accessible to solvent, in yellow color. Arrows denote the N- and C-terminal regions of both molecules.

PvRMC-MSP1 prepared in water displayed a structure pattern consisting of α-helical conformations which were revealed by the presence of two minimal ellipticity values at 208 nm and 222 nm (shown in solid black, Fig. 2A). This structure pattern could indicate that the molecule in solution stabilizes in a high percentage of alpha-helical segments. When PvRMC-MSP1 was analyzed in a stronger hydrophobic system, a 30% TFE-aqueous solution, the CD profile obtained revealed a strong anti-parallel -β- turn behavior, shown by a minimum ellipticity value at 222 nm and a maximum value at 205 nm (shown in red, Fig. 2A). It is well known that TFE and other alcohol-derived solvent systems stabilize alpha-helical structure conformations on polypeptides^29^. Remarkably our experiments showed that a preferential β–turn conformation was highly stabilized within PvRMC-MSP1 in TFE solution. This structural feature could have an impact on the functional activity of the chimeric protein *in vivo*.

Another important characteristic necessary for the functionality of PvMSP1 based proteins is the conservation of the disulfide bridges between the two EGF domains present in the 19 kDa fragment, as the proper conformation of the molecule which depends on the formation of these bridges is necessary for the production of protective antibodies^30^. To confirm that PvRMC-MSP1 maintains the MSP1_19_ fragment conformation, we used polyclonal antibodies obtained from mice immunized with PvMSP1_19_ and absorbed all the antibodies directed against the linear epitopes using an ELISA in which the antigen was PvRMC-MSP1 in a reduced state. After the absorption of the linear antibodies, we tested if the post-absorption antibodies which could then be considered “conformational” were able to recognize PvRMC-MSP1 in a reduced and a non-reduced state. We observed that the conformational antibodies recognized the protein in its conformational state but not when this protein is reduced, confirming that PvRMC-MSP1 maintains the structural elements of PvMSP1_19_ (Supplementary Figure S3).

#### CD data deconvolution

The aim of data deconvolution is to provide a quantitative estimation of the secondary structural elements, using the data determined in CD experiments, represented as the amount of protein that adopts a given structural profile. This approach allows us to analyze the secondary structural components present in the entire PvRMC-MSP1 quantitatively.

Data deconvolution was performed using three different platforms: CDsstr, Continll, and Selcon3. The PvRMC-MSP1 chimeric protein was analyzed under high hydrophilic conditions (100% water). CDsstr analysis produced a profile consisting of 53.0% α-helix, 31.6% β-strand and 15.4% random coil. Using Continll, we obtained a profile of 25.7% α-helix, 46.1% β-strand and 28.2% random coil. Selcon3 analysis produced a profile that included 25.2% α-helix, 47.6% β-strand and 27.2% random coil. Of these three algorithms, Continll and Selcon3 defined a higher percentage of β-turn structural content for PvRMC-MSP1 in water.

When PvRMC-MSP1 CD data was analyzed in a hydrophobic environment (30% TFE-aqueous solution), CDsstr analysis produced a profile consisting of 25.0% α-helix, 49.0% β-strand and 26.0% random coil. A composition of 25.7% α-helix, 46.1% β-strand and 28.2% random coil was obtained by analysis with Continll, and a profile consisting of 25.2% α-helix, 47.6% β-strand and 27.2% random coil was obtained by analysis with Selcon3. Unlike our observations under highly hydrophilic conditions, when PvRMC-MSP1 was analyzed in stronger hydrophobic conditions all algorithms were in agreement with a β-turn structural conformation. Remarkably, in both solvent systems, the amount of randomly organized structures ranged from 22.0% to 28.2% thus the whole CD profile in both cases was due to highly structured polyproteins in solution.

#### Three-dimensional structure predictive models for PvRMC-MSP1 and PvMSP1_19_ chimeric proteins

Molecular representations for protein structures, shown as colorful ribbons representing the protein backbones, allow us to infer local structural constraints in both PvRMC-MSP1 and PvMSP1_19_ (Fig. 2B). PvRMC-MSP1 displays five alpha-helical regions interconnected by more flexible beta-stranded regions. The following alpha-helical regions were identified from M_1_ to L_24_ for the first, and between F_30_ to H_49_, L_55_ to D_74_, L_80_ to S_99_ for the intermediate three. The final alpha helix is larger and closer to the C-terminal from S_105_ to N_124_, as observed in Fig. 2B.

The global 3D structure revealed an anti-parallel-β-strand region anchored to the His-rich C-terminal portion of PvRMC-MSP1, which was also evident in the CD experiments. At the *N*-terminal region of PvRMC-MSP1, where the T cell epitopes are located, the 3D structure was revealed to be highly α–helical, while the more flexible regions where the GPGPG (Gly-Pro-Gly-Pro-Gly) linkers are located are consistent with β-strand and β-turn conformations. The molecular model obtained for PvMSP1_19_ displayed a relevant content of β-strand, β-turns and antiparallel-β-strand conformations. These protein features provide high energy and flexible conformations to the protein due to the addition of a substantial number of degrees of freedom to the whole molecule (Fig. 2B).

Regarding the quality of computationally predicted protein structures, both data systems were validated by using the remote Swiss-Model server for each processed data set corresponding to PvRMC-MSP1 and PvMSP1_19_. Ramachandran plot considerations were obtained using Procheck software, which checks the stereochemical quality. For PvRMC-MSP1 (290 residues) and PvMSP1_19_ (162 residues) we obtained values of 80% and 60% respectively. These data demonstrate a minimal violation of the geometrical and stereochemical parameters in the predicted protein structures such as backbone conformation, amino acid side-chain orientation, peptide and chemical bonds length, peptide-bond angles and planar groups. Therefore, the whole molecular restraint and constraint features were in a desirable statistical range to be considered allowed conformations for both 3D molecular structures.

### Humoral immune response in mice immunized with PvRMC-MSP1

#### Anti-protein and anti-peptide antibody responses

**Figure 3:**
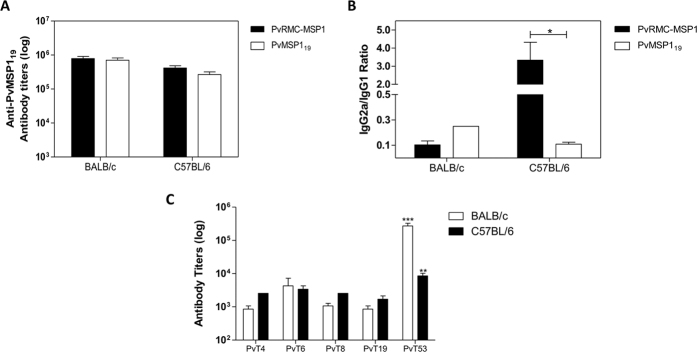
Humoral responses induced by PvRMC-MSP1 or PvMSP1_19_. (**A**) Anti-MSP1_19_ antibody titers determined in sera samples (n = 10 per group) collected 20 days after the final immunization with PvRMC-MSP1 or PvMSP1_19_ (day 60). Bars represent arithmetic mean values for each group. (**B**) Anti-PvRMC-MSP1 IgG2a/IgG1 ratio after the immunization with PvRMC-MSP1 (closed bars) and PvMSP1_19_ (open bars) sera from both BALB/c and C57BL/6 mice was obtained 20 days after the final immunization (n = 10 per group), *p = 0.0104 by Student’s t test. (**C**) Recognition of the promiscuous T cell epitopes present in PvRMC-MSP1 by BALB/c (open bars) and C57BL/6 (closed bars) mice 20 days after the final immunization. **p < 0.01 ***p < 0.001 by one-way ANOVA with Bonferroni posttest.

After the third and final immunization with PvRMC-MSP1, antibody titers against PvMSP1_19_ had a mean titer of 7.9 × 10^5^ in BALB/c and 4.1 × 10^5^ in C57BL/6 mice. These titers were similar to those obtained after the immunization with the native PvMSP1_19_ (Fig. 3A). We measured the IgG1 and IgG2a levels to determine if the inclusion of promiscuous T cell epitopes modified the Th1 or Th2 response patterns. In BALB/c mice, similar IgG2a/IgG1 ratios were observed between the groups of mice immunized with PvRMC-MSP1 or PvMSP1_19_, with both ratios indicating a predominantly Th2 response. Meanwhile, C57BL/6 mice immunized with PvRMC-MSP1 exhibited higher IgG2a/IgG1 ratios when compared to mice immunized with PvMSP1_19,_ demonstrating that immunization of C57BL/6 mice with PvRMC-MSP1 skewed the response towards a Th1 phenotype, which was not the case for vaccination with PvMSP1_19_ (Fig. 3B). We also measured the avidity to PvMSP1_19_ in mice immunized with PvRMC-MSP1, as an indicator of the antibodies quality. In BALB/c mice, an average of 3.3 M of thiocyanate was required to dissociate 50% of the bound anti-PvMSP1_19_ antibodies elicited by immunization, corresponding to an avidity index of 0.71. Antibodies elicited in C57BL/6 mice required an average of 4.5 M of thiocyanate for 50% dissociation, exhibiting an avidity index of 0.87.

We also tested the antibody responses against the amino terminal promiscuous T cell epitopes included in PvRMC-MSP1. Immunization with PvRMC-MSP1 elicited significantly higher antibody titers against PvT53 when compared to the antibody responses elicited by other epitopes in both BALB/c and C57BL/6 mice (Fig. 3C).

We tested sera samples by immunofluorescence assay against *P. vivax* infected erythrocytes, to assess the reactivity of the antibodies induced by immunization with PvRMC-MSP1 against the native protein. Both mice strains recognized blood stages with a pattern of fluorescence reported for PvMSP1 (Fig. 1D) with no significant differences in antibody titers (p = 0.07). Nonetheless, C57BL/6 mice showed a trend for higher antibody titers (Geometrical mean 2.1 × 10^5^) than BALB/c mice (Geometrical mean 5.1 × 10^4^).

#### Native and cross-reactive antibody responses

The *P. vivax* T cell epitopes used in PvRMC-MSP1 were selected based on their ability to function as promiscuous epitopes and the high frequency of specific T cells present in the peripheral blood of individuals naturally infected with vivax malaria^24^. Given the phylogenetic similarities between *Plasmodium vivax* and the simian parasites *P. cynomolgi* and *P. coatneyi,* and considering the potential of using these parasites as surrogate models to test protective efficacy in rhesus macaques, we decided to assess if sera samples from BALB/c and C57BL/6 mice immunized with PvRMC-MSP1 cross-react with *P. coatneyi* MSP1. PvRMC-MSP1 promiscuous T cell epitopes had between 70 and 95% homology for the same MSP-1 sequences in *P. coatneyi* and between 85 and 95% for *P. cynomolgi* MSP-1 (Table 1, Supplementary Table S1). Antibodies derived from both strains of immunized mice recognized *P. coatneyi* blood stage parasites (Fig. 1D), providing additional evidence that PvRMC-MSP1 induces antibodies that are capable of recognizing the naturally derived MSP-1 protein. The PvRMC-MSP1 T cell epitopes can act as universal epitopes given their structural features, including a large aromatic or hydrophobic residue in position 1 (Y, F, W, L, I, V, M) and a small, noncharged residue in position 6 (S, T, C, A, P, V, I, L, M) (Table 1 underlined)^31^. This biochemical characteristic known as the P1-P6 motif was conserved for both *P. coatneyi* and *P. cynomolgi* in four of the five promiscuous T cell epitopes and also in the MSP1_33_ fragment used in our chimeric protein (Supplementary Table S1). We also determined the protein sequence identity of promiscuous T cell epitopes in comparison to five representative *P. vivax* strains (i.e. Brazil I, India VII, Salvador I, North Korea, Mauritania I). The epitopes exhibited a protein identity that ranged from 90 to 100%. Relevantly, none of the amino acid changes is located in the P1-P6 motif (Supplementary Table S2), confirming the highly conserved nature of these epitopes.

### Cellular immune responses in mice immunized with PvRMC-MSP1

#### Cellular responses against PvMSP1_19_

**Figure 4:**
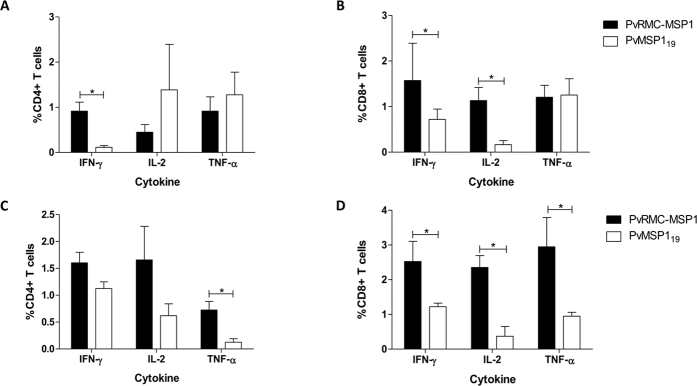
T cell responses after immunization with PvRMC-MSP1 or PvMSP1_19._ Five Days after the final immunization spleens from BALB/c or C57BL/6 mice immunized with either PvRMC-MSP1 (n = 5 per strain) or PvMSP1_19_ (n = 5 per strain) were processed and stimulated with PvMSP1_19_ and the frequency of cytokine-secreting T cell assessed. Top Panel. CD4+ (**A**) and CD8+ (**B**) cytokine secreting T cells in C57BL/6 mice. Bottom Panel. CD4+ (**C**) and CD8+ (**D**) cytokine secreting T cells in BALB/c mice. Results are presented after background subtraction using three technical replicates per sample. Statistical analysis was done using Mann-Whitney test. *p < 0.05.

At day five after the final immunization, flow cytometry and intracellular cytokine staining were performed to characterize the T cell reactivity induced by vaccination with PvRMC-MSP1 or the native PvMSP1_19,_ following *ex vivo* stimulation of splenocytes with PvMSP1_19_. In C57BL/6 mice, there was a significantly higher frequency of IFN-γ secreting cells in response to PvMSP1_19_ by both CD4+ (p = 0.047) and CD8+ (p = 0.024) T cells in the group immunized with PvRMC-MSP1 when compared to the mice immunized with the native PvMSP1_19_ (Fig. 4A,B). In BALB/c mice, the frequency of IFN-γ secreting cells was significantly higher in CD8+ T cells (p = 0.037) but not in CD4+ T cell population (p = 0.108) (Fig. 4C,D). There were no significant differences in the frequency of IFN-γ secreting cells between the strains in either CD4+ (p = 0.068) and CD8+ (p = 0.395) T cells. On the frequency of IL-2 and TNF-α secreting CD4+ T cells, we observed significant differences in the production of TNF-α in BALB/c mice (p = 0.024), but there were no other differences between the groups or the strains after the stimulation with PvMSP1_19_. Importantly, in both strains, the CD8+ T cells were able to produce IL-2 in response to PvMSP1_19_ in a significantly higher proportion after the immunization with PvRMC-MSP1.

#### Cellular responses against PvRMC-MSP1

**Figure 5:**
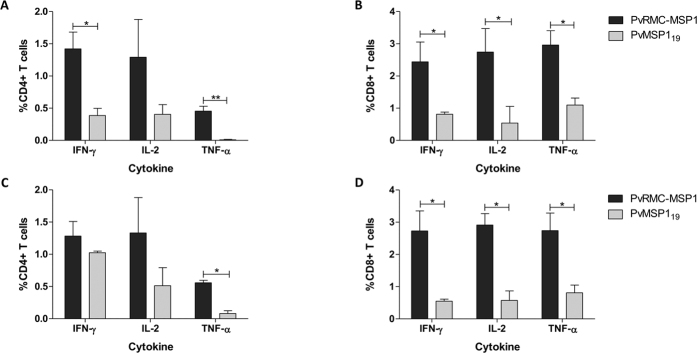
T cell responses to the different components of PvRMC-MSP1 in BALB/c mice. Five days after the final immunization spleens from BALB/c mice immunized with either PvRMC-MSP1 (n = 5) or PvMSP1_19_ (n = 5) were processed and stimulated with peptide pools representing the different components of PvRMC-MSP1 and cytokine-secreting T cells assessed. Top Panel. CD4+ (**A**) and CD8+ (**B**) cytokine secreting T cells after stimulation with peptide pools representing the promiscuous T cell epitopes present in PvRMC-MSP1. Bottom Panel. CD4+ (**C**) and CD8+ (**D**) cytokine secreting T cells after stimulation with peptide pools representing the PvMSP1_19_ protein fragment present in PvRMC-MSP1. Results are presented after background subtraction using three technical replicates per sample. Statistical analysis was done using Mann-Whitney test. *p < 0.05 **p < 0.01.

We assessed the cellular response to the different components of PvRMC-MSP1 using *ex vivo* stimulation with peptide pools representing the promiscuous T cell epitopes (Fig. 5A,B) or PvMSP1_19_ (Fig. 5C,B), (Supplementary Table S3). Following stimulation with the peptides representing the promiscuous T cell epitopes, BALB/c mice immunized with PvRMC-MSP1 showed a significantly higher proportion of IFN-γ and TNF-α secreting CD4+ and CD8+ T cells when compared to mice immunized with PvMSP1_19_ (Fig. 5A,B). Although the percentage of IL-2 secreting cells was higher in the CD4+ T cells from the PvRMC-MSP1 immunized mice, this difference was not significant. In contrast, CD8+ T cells from PvRMC-MSP1 displayed a greater proportion of IL-2 producing cells when compared to the cells from mice immunized with the native PvMSP1_19_. The frequency of IFN-γ and IL-2 secreting CD4+ T cells after the stimulation with peptide pools representing PvMSP1_19_ were not significantly different between the immunization groups in BALB/c mice (Fig. 5C). Nonetheless, the responses were higher in the PvRMC-MSP1 group (Fig. 5C). Importantly after the stimulation with the peptide pools representing PvMSP1_19,_ there were a significantly higher proportion of CD8+ T cells able to produce all the analyzed cytokines in the PvRMC-MSP1 immunization group confirming that unlike the immunization with the native PvMSP1_19_ our chimeric protein improved the recognition of PvMSP1_19_ by CD8+ T cells. We obtained similar results in C57BL/6 mice (Supplementary Figure S4).

### Seroepidemiological studies

#### Frequency and magnitude of Human IgG immune response against PvRMC-MSP1

**Figure 6:**
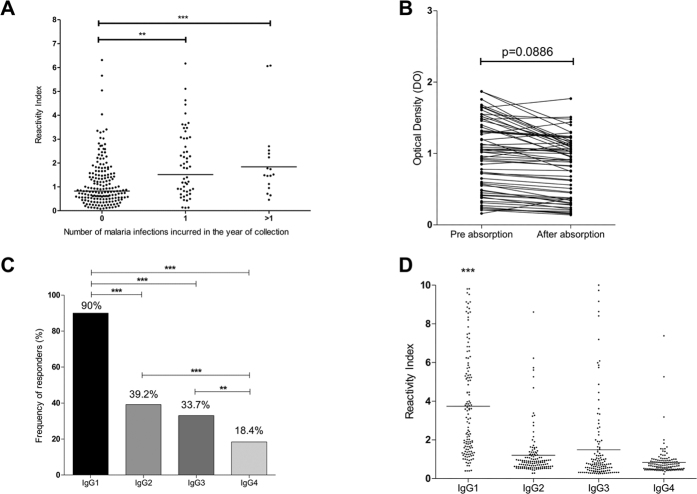
Antibody recognition of PvRMC-MSP1 by individuals naturally exposed to malaria. (**A**) Reactivity Index in the studied population to the recombinant chimeric protein PvRMC-MSP1. The geometric mean of the IgG reactivity index against PvRMC-MSP1 was significantly higher in people with 1 or more malaria infections in the year of collection. **p < 0.01 ***p < 0.001 by Mann-Whitney test. (**B**) IgG titers against PvRMC-MSP1 presented as optical densities after absorption of the antibodies against the (NANP)_6_ peptide. No significant differences were observed between the optical densities before and after absorption. (**C**) Frequency of IgG subclass responders to the recombinant chimeric protein PvRMC-MSP1 among the IgG responders. A χ^2^ test was performed to determine the differences between the frequency of IgG1 responders compared with all others IgG subclasses. **p < 0.01 ***p < 0.001. (**D**) Reactivity Index by IgG subtype of the samples against PvRMC-MSP1. ***p < 0.001 by one-way ANOVA with Bonferroni’s posttest.

To evaluate the frequency of antibody responses to PvRMC-MSP1 in a naturally exposed population, plasma samples collected from 258 individuals living in two areas where *P. vivax* accounts for more than 70% of the clinical cases of malaria were assessed by ELISA. We collected blood samples from rain forest natives who have resided in the malaria-endemic region for over 25 years (Ribeirinha) or individuals who were transmigrants from several non-endemic areas of Brazil, living in the malaria endemic area for the last decade (Colina). The prevalence of anti-PvRMC-MSP1 antibodies in the populations studied was 50.4% (130/258 individuals). There was a high total IgG reactivity against our chimeric protein as shown by the reactivity indexes (RI) ranging from 0.08 to 12.3 (Fig. 6A).

PvRMC-MSP1 contains six copies of the *P. falciparum* NANP repeat sequence as a C-terminal tag for characterization and purification purposes. Since the sera samples collected in the present study belong to individuals living in an area where *P. falciparum* also occurs, it is possible that antibody responses to the CSP repeats can also account for the anti-PvRMC-MSP1 reactivity. To study the contribution of antibodies to *P. falciparum* CSP repeats in the response to PvRMC-MSP1, we performed absorption ELISA experiments. PvRMC-MSP1-positive antibody samples from 63 randomly selected individuals were pre-incubated with an (NANP)_6_ synthetic peptide before the evaluation of IgG reactivity against PvRMC-MSP1. These experiments showed that only 31.7% of the samples were positive against (NANP)_6_ with a reactivity index (RI) ranging from 0.4 to 2.690 (median 0.85) (Supplementary Figure S5). We compared the optical densities of the samples that reacted against PvRMC-MSP1 before and after the absorption procedure and found no significant differences (Fig. 6B). Although we cannot guarantee that this process removed all anti-NANP antibodies, the RI against (NANP)_6_ after absorption presented a very significant decrease (p < 0.0001). Moreover, all 20 individuals who were NANP responders before the absorption, became non-responders (Supplementary Figure S5). The results confirm that the antibody reactivity reported by PvRMC-MSP1 is not affected by the (NANP)_6_ tag.

Antibodies from responders were further analyzed to determine the IgG subclass profile (Fig. 6C). The cytophilic IgG1 subclass was the predominant type being present in 90% of the samples (Fig. 6C) a proportion that was significantly higher when compared to the other subclasses (p < 0.001 by Χ^2^ test). When we analyzed the levels of specific antibodies as a reactivity index, there was a noteworthy predominance of IgG1 antibodies (Fig. 6D).

#### Relationship between malaria exposure and PvRMC-MSP1 response

To assess whether epidemiological factors influence the naturally acquired immune response against PvRMC-MSP1, we correlated different parameters of the population with the reactivity indexes of total IgG and the IgG subclasses (Table 2). We did not observe a correlation between the age, the time of residence in the malaria endemic area, or the time of residence in Rondonia (the population with the highest endemicity of those studied). To assess the effect of malaria history in the immune response against PvRMC-MSP1, we correlated the number of past infections and the months since the previous infections. We observed that the number of malaria infections contracted in the past showed a significant direct correlation with antibody response to PvRMC-MSP1 (r = 0.1750, p = 0.0052), indicating an additive effect of the specific immune response acquired. Most importantly the anti-PvRMC-MSP1 antibodies were negatively correlated with the months since the previous infections suggesting a potential role for the anti-PvRMC-MSP1 antibodies in protection.

#### Influence of HLA in IgG response

To determine if antibodies directed against PvRMC-MSP1 were genetically restricted, we evaluated the influence of HLA-DRB1* and HLA-DQB1* alleles and haplotypes on the naturally acquired IgG response to PvRMC-MSP1. Although 49.9% of the population did not present detectable titers of antibodies against PvRMC-MSP1, there does not appear to be a genetic restriction to this antigen, since we did not observe any association between the HLA DRB1* or DQB1* alleles and the antibody response to PvRMC-MSP1 (Supplementary Table S4).

## Discussion

The design of an effective malaria vaccine has proven to be a formidable research challenge. As a target, MSP1 offers appealing features as it is expressed in both the hepatic and the erythrocytic stages of infection^32^. Although antibodies against this antigen are involved in protection^9^,^10^, the protective role of the cellular immune responses induced by MSP1 is still unclear. MSP1_19_ is the protein fragment involved in the humoral response, but the cellular response induced by the whole MSP1 protein seems to target the MSP1_33_ fragment^33^. While CD8+ T cell epitopes have been described in *P. falciparum* MSP1_19_, a cellular response against this antigen has not been achieved, even when viral vectors are used to deliver this fragment^34^.

Here, we report the design and characterization of a Recombinant Modular Chimera (RMC) based on PvMSP1 in which defined cognate promiscuous T cell epitopes were genetically linked through GPGPG spacers to enhance the immunogenicity of PvMSP1_19_. We have previously used this approach in proof of concept studies using synthetic peptides or recombinant proteins^26^,^27^,^35^. CD data support the rationale for using this protein topology. The Ramachandran plot is used as a way to visualize the backbone dihedral angles ψ against φ allowed for amino acid residues in a protein structure. The plot, therefore, shows the different segments that correspond to “core regions” representing the most favorable combinations of φ-ψ values. Values higher than 60% are statistically relevant and are considered “favored or allowed conformations”^36^. In our particular case, 80% and 60% represent the stable protein conformations in solution for both the PvRMC-MSP1 construct and for PvMSP1_19_, respectively. Hence, the presence of T cell epitopes in alpha-helical conformation within PvRMC-MSP1 would allow stable MHC – antigen – TCR interactions, leading a hydrogen bonding network for the immuno-dominant ternary complexes.

The molecular modeling of PvRMC-MSP1 also validated the reported structure of the *P. vivax* MSP-1_19_^7^. Although the N- terminal domain of the chimeric protein contains distinct structural elements, the C-terminal domain assumes the closely packed U-shape conformation characteristic of the *P. vivax* MSP1_19_. This structural feature includes two epidermal growth factor (EGF)-like domains, present in tandem conformation (Fig. 2B). Crystallographic data available for *P. cynomolgi*^37^ and *P. knowlesi*^38^ defined the classical array of disulfide bonds in the second EGF-like domain between cysteines 1–3, 2–4, and 5–6 with a second disulfide bond missing in the first EGF-like domain. EGF-like domains present within PvRMC-MSP1 are located between amino acids M_167_ and V_213_, and T_214_ and S_258_ and contain two anti-parallel beta-strand pairs (Fig. 2B). Relevantly, PvRMC-MSP1 induced antibodies that are able not only to recognize the recombinant *P. vivax* MSP1_19_ but the native protein on the parasite surface, showing that the recognized epitopes are accessible.

As expected, immunization with PvRMC-MSP1 elicited antibody titers higher than 1 × 10^5^ in two different mouse strains after three immunizations. These antibodies were able to recognize our vaccine candidate, a recombinant protein representing PvMSP1_19_ and the native protein in *P. vivax* infected RBCs. Total IgG antibody titers induced by PvRMC-MSP1 are similar to those reported for a synthetic protein expressed in *Saccharomyces cerevisiae* formulated in Freund’s adjuvant^39^. The formulation of PvRMC-MSP1 in Montanide ISA 51 supports the fact that the immunogenicity of our formulation is dependent on the inclusion of the T cell epitopes rather than on the adjuvant^22^,^39^.

The immune response was skewed towards a Th1 profile following immunization of C57BL/6 mice with PvRMC-MSP1, as we observed IgG2a/IgG1 ratios higher than one, which was not the case in other PvMSP1 vaccine candidates^39^,^40^,^41^,^42^,^43^. Although high levels of IgG2a were also detected in BALB/c mice, the IgG2a/IgG1 ratio suggested a Th2 biased response, which is expected since the genetic regulation of the immune response the BALB/c strain favors the production of IL-4 over IFN-γ^44^. Even when viral vectors are used to present PfMSP1, the IgG2a/IgG1 ratios in BALB/c are still indicative of a Th2 response^34^. Our results suggest that the help provided by the promiscuous T cell epitopes enhances the production of cytophilic antibodies. The production of cytophilic antibodies by PvRMC-MSP1 is encouraging since the inhibition of red blood cell invasion is correlated with the ability of cytophilic antibodies directed against MSP1 to interact with complement^11^. In addition to the ability of PvRMC-MSP1 to elicit the production of cytophilic antibodies in mice, sera samples from humans naturally exposed to *P. vivax* had a high prevalence of cytophilic IgG1 antibodies that recognize PvRMC-MSP1. The quality of the antibody response elicited by immunization was also confirmed by the assessment of the higher avidity of anti-PvRMC-MSP1 antibodies in C57BL/6 mice. Similarly, phase I clinical studies carried on with a long synthetic peptide based on *P. falciparum* MSP3 have shown that anti-parasite antibody-dependent cellular inhibition is related to the antibodies avidity^45^. These results demonstrate that immunization with PvRMC-MSP1 could achieve a high avidity cytophilic IgG response that is correlated with protection.

Consistent with the high homology (>70%) between the promiscuous T cell epitopes and the MSP_19_ fragment included in PvRMC-MSP1 and the simian malaria species *P. coatney*i and *P. cynomolgi*, we observed that antibodies induced by PvRMC-MSP1 were able to recognize *P. coatneyi* blood stage parasites by IFA. The antibodies recognition of other species confirms that the promiscuous *P. vivax* T cell epitopes included in PvRMC-MSP1 are conserved in the simian *Plasmodium* species and preserve their biochemical features. This feature also demonstrates the potential of using *P. cynomolgi* or *P. coatneyi* in rhesus macaques as a surrogate model to test efficacy as *P. vivax* appears to be a species derived from the *Macaca* lineage of simian parasites^46^.

Preclinical trials in NHP have been reported for PvMSP1 based vaccines. PvMSP1_19_ expressed in *S. cerevisiae* linked to two tetanus toxoid promiscuous T-helper epitopes^21^ induced partial protection in *Saimiri boliviensis* monkeys. However, the immunogenicity of the protein was highly dependent on the adjuvant used in the formulation^21^,^47^. This effect was also observed when an MSP1_19_ protein associated to two pan allelic CD4+ T cell epitopes was used in *Callithrix jacchus*, with no evident boosting effect induced by the associated T cell epitopes^22^. Studies using MSP1_33_ in *Aotus* monkeys have shown a protective efficacy between 50 to 80% according to the fragment of the protein used. However, these studies used Freund’s adjuvant, a formulation that is not suitable for human use^23^,^48^. In another study, recombinant PvMSP1 formulated in Montanide ISA 720 was also able to protect *Aotus* monkeys from an infectious challenge reducing the parasitemia levels in comparison with control monkeys^49^. These studies demonstrate that achieving protection with PvMSP1 is a feasible goal; but further development is required to obtain a safe, highly immunogenic formulation.

Studies with recombinant PvMSP1_19_ have shown that this protein is not processed by DCs since the antigen is not soluble and remains attached to the parasite during the ring stage following RBC invasion^50^. In rodent models the conserved disulfide bridges, the main structural feature of the native proteins, impede antigen processing thereby preventing the generation of an effective cellular response^14^. Despite this, PvRMC-MSP1 was able to induce strong CD4+ and CD8+ T cells in two different strains of mice. To our knowledge, this is the first evidence that a recombinant subunit protein can induce both CD4+ and CD8+ T cells able to recognize PvMSP_19_. Three factors could explain the improved cellular immunogenicity of our construct: (1) The defined *P. vivax* promiscuous T cell epitopes, specially PvT53 and PvT19, have been shown to increase the immunogenicity of diverse antigens, as closely related as the *P. falciparum* CSP (NANP)_3_ repeats^24^, or as far as B cell epitopes of the Tau and Amyloid-Beta proteins related to Alzheimer disease^51^. (2) We have shown the ability of our chimeric proteins with the topology used for the design of PvRMC-MSP1 to enhance dendritic cells uptake and maturation^52^. (3) Our promiscuous T cell epitopes resemble a family of compounds that are known as cell penetrating peptides (CPPs)^52^. CPPs are linear sequences ranging between 10 and 30 amino acids that contain positively charged residues (W, R, or F) and can deliver a diverse repertoire of bioactive molecules^53^. Peptide uptake is enhanced by the interaction of positively charged residues with negatively charged membranes, resulting in endocytosis by antigen presenting cells^54^.

The CD4+ T cells induced by immunization with PvRMC-MSP1 produced higher cytokine levels than those induced by PvMSP1_19_, suggesting that CD4+ T cell activation by PvRMC-MSP1 allows these cells to recognize both MSP1_19_ and other conserved regions through the whole MSP1 sequence and produce a balanced cytokine response, an effect not achieved by the native PvMSP1_19_. CD4+ T cell activation has been correlated with protection against blood stage *P. falciparum* malaria in humans undergoing a controlled challenge under chloroquine prophylaxis^17^. The ability of CD4^+^ T cells to produce several cytokines has been proposed as a marker of their effector and long-term memory potential^55^. In the murine *P. chabaudi* model, effector memory T cells are related with protection^56^, while the antibody response against MSP1_19_ induced by central memory T cells is a vital correlate of protection^57^,^58^. Therefore, the balanced CD4+ activation profile obtained with PvRMC-MSP1 is a desirable characteristic in a blood stage vaccine candidate.

Furthermore, PvRMC-MSP1 was able to induce cytokine-producing CD8+ T cells able to recognize the PvMSP_19_ antigen and peptide pools representing the entire sequence, confirming the CD8+ recognition of PvMSP_19_. Predictions by the artificial neuronal network algorithm provided by the IEDB server^59^,^60^,^61^,^62^,^63^,^64^,^65^,^66^,^67^ suggest the presence of potential H2-K^b^, H2-D^d^ and H2-L^d^ CD8+ T cell epitopes in the N-terminal and central regions of the recombinant protein. In humans, the same algorithm predicts a much larger number of CD8+ T cell epitopes scattered along the whole length of the recombinant PvRMC-MSP1 protein. Most of the predicted CD8+ T cell epitopes are restricted by the frequently found HLA-A*, few by HLA-B* and almost none by HLA-C* alleles (Supplementary Figure S6). A cytotoxic effect against blood stage antigens by CD8+ T cells mediating protection have been demonstrated in murine models^19^. In humans, *P. falciparum* vaccine candidates have shown that a CD8+ T cell response recognizing PfMSP1_42_ can increase the patency time and reduce the infective load that passes from the liver into the blood, as MSP1 is expressed late in the liver stage cycle^20^. Consequently, a *P. vivax* vaccine able to induce CD8+ T cells like PvRMC-MSP1 should be considered a strong candidate for inducing multi-stage immunity.

Although several studies have shown that CD8+ T cells able to recognize blood stage *Plasmodium* antigens are related with protection, studies using the murine *P. berghei* ANKA model have suggested that CD8+ T cells are also involved in the pathogenesis of cerebral malaria. Perforin-mediated killing by CD8+ T cells^68^ of the endothelial cells in the cerebral microvasculature damages the blood-brain barrier and is one of the most important events on the cerebral malaria pathogenesis^69^. Consistent with this finding, the depletion of CD8+ T cells reduces murine mortality^70^. Importantly, the CD8+ T cells induced by the infection with *P. berghei* can be antigen-specific and non-specific. The antigen non-specific CD8+ T cells can proliferate, show an activation phenotype, express granzyme B and gain CTL function^71^. Therefore, the pathogenesis of cerebral malaria is not dependent on the recognition of a single blood stage antigen by CD8+ T cells, but rather is a multifactorial event induced by the malarial infection. As such it cannot be considered that CD8+ T cells induced by vaccination would enhance the processes leading to cerebral malaria.

The 50.4% frequency of responders to PvRMC-MSP1 among the studied population is similar to other MSP1 seroprevalence studies in different endemics areas of Brazil^72^,^73^,^74^. Other studies have proposed that only antibodies from patients with active *P. vivax* infection were able to recognize PvMSP-1^49^, suggesting that the *P. vivax* natural infection can induce short-lived antibodies not related to protection. In contrast, PvRMC-MSP1 recognition was present in patients that did not have active malaria, which could be an effect of our vaccine design strategy as we use well-defined T and B cell epitopes to avoid the inclusion of regions of low immunogenic potential. We have previously demonstrated that our constructs are better recognized by naturally infected individuals when compared to native proteins and can circumvent the HLA restriction associated to these antigens^27^,^35^. These characteristics make our proteins not only desirable for vaccination but also useful for seroepidemiological studies.

We observed that naturally acquired antibodies against PvRMC-MSP1 exhibited a cytophilic profile. In *P. falciparum* infection, human cytophilic antibodies against MSP1 and MSP2 have been related to inhibition of the red blood cell invasion^11^. In *P. vivax*, an antibody profile similar to the one obtained in our study was found to be related to low parasitemias^75^,^76^. Anti-PvRMC-MSP1 antibodies had a higher prevalence of cytophilic antibodies when compared with previous reports from symptomatic patients^77^, with an IgG1 prevalence of 90% and an IgG3 prevalence of 33.7%. Evidence of the possible PvRMC-MSP1 protective effect is also observed with the correlation of the production of antibodies able to recognize this vaccine candidate and the months passed since the previous malaria episode.

There were no associations between the PvRMC-MSP1 responses with the HLA alleles present in the population studied. We have shown that our chimeric proteins can circumvent genetic restriction to the recognition of pre-erythrocytic and erythrocytic *P. vivax* antigens^27^,^35^. It is important to test this protein in non-human primates from different genetic backgrounds to confirm the induction of non-genetically restricted immune responses, a highly desirable characteristic for a *P. vivax* malaria vaccine.

The individual linkage of promiscuous T cell epitopes present in PvRMC-MSP1 to the previously defined *P. falciparum* B cell epitope (NANP)_6_ that constitute the repeat motif of the central region of the circumsporozoite protein was able to induce protective antibodies able to block *P. falciparum* invasion of hepatic cells *in vitro*^24^. This epitope is also present in PvRMC-MSP1, and we observed that ~30% of the individuals in our seroprevalence study have antibodies to the (NANP)_6_ repeats. The lower prevalence of the response against *P. falciparum* CS repeats was expected since the prevalence of *P. falciparum* is lower in the studied population^78^. However, these results show that a multispecies malaria vaccine is feasible and should be considered in the design of future RMCs.

In conclusion, we report a PvMSP1 based chimeric protein, PvRMC-MSP1, designed to express several amino terminal cognate promiscuous T cell epitopes. PvRMC-MSP1 was able to induce cytophilic antibodies and previously unreported CD4+ and CD8+ T cell responses against PvMSP1_19_ in mice. The high immunogenicity of PvRMC-MSP1 confirms our previous reports concerning the benefits of designing chimeric proteins using such topology. Antibodies also recognized the chimeric protein in plasma samples collected from naturally exposed individuals irrespective of their HLA haplotype with an immunoglobulin profile that is related to protection. These characteristics make PvRMC-MSP1 a highly promising synthetically designed vaccine candidate that warrants further investigation in clinical models.

## Methods

### Design and biochemical characterization of the *P. vivax* chimeric MSP-1

The 861 bp *pvrmc-msp1* gene was codon optimized and synthesized by Geneart (Regensburg, Germany) (Fig. 1A,B). The *pvrmc-msp1* synthetic gene is based on the reference *P. vivax* Belem sequence (GenBank: XP_001614842.1) and encodes a chimeric protein that includes: (1) Met-Ala on the N-terminus to provide the start signal and decrease degradation in *E. coli*, respectively. Two additional amino acids, V-D, introduced downstream as part of the cloning strategy (Fig. 1A). (2) Five promiscuous T cell epitopes linked in tandem, [PvT4 (N_78_-L_97_), PvT6 (F_118_-H_137_), PvT8 (L_158_-D_177_), PvT19 (L_378_-S_397_) and PvT53 (S_1058_-N_1077_)], which were previously reported by our group using peptide binding competition assays^24^. (3) An extended version of the *P. vivax* MSP1_19_ protein fragment sequence, which includes two T helper epitopes present in the MSP1_33_ protein fragment. (4) Six copies of the *P. falciparum* circumsporozoite protein repeat region, (NANP)_6_, were included at the C-terminus for biochemical characterization of antigenic integrity and to provide an optimal affinity purification tag. (5) GPGPG spacers were inserted between the described sequences to enhance antigen processing and the stability of the protein.

The *pvrmc-msp1* synthetic gene was digested with restriction enzymes *Nco I* and *Xho I*, and subsequently cloned into the pET24d(+) plasmid, which results in the expression of the protein with a C-terminal (His)_6_-tag. The plasmid containing the chimeric construct was then transformed into *E. coli BL21* (DE3) cells (Novagen, Madison WI) and protein expression was induced by the incubation with one mM IPTG for 3 hours.

The resulting 290 amino acid protein, including the 6x His-tag and two additional amino acids (L-E) introduced upstream from the His-tag as part of the cloning strategy, was purified with a Ni-NTA affinity column according to the manufacturer’s protocol (Qiagen, Valencia, CA). After initial metal chelate purification, the recombinant construct was purified using analytical gel filtration chromatography. Analyses by SDS-PAGE showed a single band of the apparent mobility of ~31 kDa. The protein was further purified by size-exclusion chromatography performed on an FPLC instrument (AKTA prime Plus, GE healthcare) using a Sephadex G-75 column.

The procedures to produce the synthetic gene encoding the *P. vivax* MSP1_19_ are identical to those described for the production of the *P. yoelii* MSP1_19_ protein^9^, using the *pvrmc-msp1* synthetic gene described above and PCR amplification. PvMSP1_19_ only contains the extended version of the *P. vivax* MSP1_19_ protein fragment and the corresponding carboxyl terminal tags. Analyses by SDS-PAGE of the 162 amino acid protein showed a single band of apparent mobility of ~22 kDa (Supplementary Figure S2).

The PvRMC-MSP1 protein was analyzed by sodium dodecyl sulfate-polyacrylamide gel electrophoresis (SDS-PAGE) under non-reducing conditions on 4–20% polyacrylamide gels (Lonza, Allendale, NJ). Following electrophoresis, western blots were performed by blotting the proteins onto nitrocellulose membranes according to standard procedures, as previously described^9^,^26^. The nitrocellulose membranes were incubated with sera samples from mice immunized with one the following synthetic peptides representing: (1) the T cell epitope PvT8 L_158_-D_177_; (2) the T cell epitope PvT19 L_378_-S_397_; (3) the T cell epitope PvT4 N_78_-L_97_; or (4) the monoclonal antibody 2A10, which recognizes the C-terminal tag (NANP)_6_, obtained from the Malaria Research and Reference Reagent Resource Center (MR4, ATCC Manassas, VA)], (Fig. 1C). Endotoxin levels were evaluated using the E-Toxate Kit (Limulus amebocyte lysate), following the manufacturer’s protocol (Sigma-Aldrich, St. Louis, MO).

### Synthetic Peptide Library

A library of 61 15-mer synthetic peptides, overlapping by 11 residues each and spanning the complete PvRMC-MSP1 chimeric protein sequence without tags, was synthesized commercially using the multiple solid-phase technique (Sigma-Aldrich, St. Louis, MO). The peptide pools were used to characterize cellular reactivity, with one set representing the sequence of the cognate T cell epitopes derived from the MSP1 structure included in our chimeric construct, and the other set representing the complete amino acid sequence of the MSP1_19_ protein fragment (Supplementary Table S3).

### Circular dichroism experiments used to determine the secondary structural elements in PvRMC-MSP1

The secondary structural elements present in the chimeric PvRMC-MSP1 protein were analyzed using a Jasco J-810 spectropolarimeter. The following conditions were used: A 5 × 10^−6^ M protein stock solution was prepared by dissolving 0.20 mg of each protein in one of two solvent solutions, either distilled deionized water or HPLC degree water-acetonitrile mixture in a 1:1 ratio. The solvent selected according to the solubility of each peptide. Following preparation of the peptide stock solution, 10 μL of the compound stock was mixed with 300 μl of 2,2,2-trifluoroethanol (TFE) in a 1.5 mL Eppendorf tube, 690 μL of H_2_O was added to bring the volume up to 1 mL. This mixture was then homogenized and transferred to a quartz cell. The circular dichroism spectrum was then recorded between a wavelength range of 190 and 260 nm.

The circular dichroism spectral data was processed using the spectropolarimeter software which subtracts the spectra from the blank solution (30% TFE in water) from the raw data and the resulting spectrum submitted to smoothing for a proper presentation. In addition, the spectropolarimeter software was used to processed the data obtained from units of millidegrees (mdeg) to molar ellipticity. The results are expressed as mean residue ellipticity [θ], with units of degrees cm^2^ mol^−1^, according to the [θ] = θ_λ_/(100*lcn*) function where θ_λ_ is the measured ellipticity, *l* is the optical path-length, *c* is the polypeptide concentration, and n is the number of amino acid residues in the sequence as described elsewhere^28^,^79^.

### CD data deconvolution process

Using the whole data set obtained for each compound molar ellipticity values, each wavelength of 0.2 nm are submitted to a subsequent deconvolution process using CDsstr^80^, Continll^29^ and Selcon3^81^ algorithms of CDPro (http://lamar.colostate.edu/~sreeram/CDPro/main.html). These algorithms allow for estimation of the secondary structural elements given experimental conditions for each polypeptide analyzed and presents the data as the percentages of α-helices, β-strands or random conformations (random coil) in their corresponding specific proportions. The secondary structure elements for alpha-helical conformations can be obtained by adding H(r) to H(d) values. Similarly, beta-strand elements can be deduced by adding S(r) to S(d) plus Trn (reverse turns) values and randomly organized elements are designed as Unrd (not-readable).

### Molecular models for PvRMC-MSP1 and PvMSP1_19_ predictions and structure quality assessment

Data for the PvRMC-MSP1 and PvMSP1_19_ protein sequences, in FASTA format, was submitted to the I-Tasser remote server (http://zhanglab.ccmb.med.umich.edu) for constructing possible 3D models^82^,^83^,^84^,^85^. The data sets were coded S198249 for PvRMC-MSP1 and S198411 for the PvMSP1_19_ fragment. For each protein, the top five most probable three-dimensional models of the protein sequences were obtained and their coordinates presented in protein data bank (PDB) format. Subsequent validation of the quality of the most probable three-dimensional structures was performed using the Swiss-model server (http://swissmodel.expasy.org) by inputting the previously obtained data sets from each molecule. As part of the quality criterion, the Q-mean index was also determined using the Swiss-model Q-mean server tool^86^,^87^,^88^,^89^.

### Three-dimensional protein prediction images

Molecular modeling was performed using VMD 1.8.6 software released from the Theoretical and Computational Biophysics Group at the University of Illinois at Urbana-Champaign (http://www.ks.uiuc.edu/Research/vmd/vmd-1.8.6)^90^,^91^.

### Mice

All animal experiments and procedures were performed in accordance with guidelines and approved by the Emory University’s Institutional Animal Care and Use Committee. Female BALB/c (H-2^d^), and C57BL/6 (H-2^b^) mice, 6 to 8 weeks of age, were purchased from Charles River (Wilmington, MA). The mice were immunized subcutaneously on days 0, 20 and 40, in the base of the tail and the interscapular area, using 20 μg of PvRMC-MSP1 or PvMSP1_19_ proteins emulsified in Montanide ISA 51 (Seppic, Fairfield, NJ). Mice in the control groups received PBS alone emulsified in the same adjuvant.

### ELISA assays using murine sera samples

Antibodies elicited by immunization with PvRMC-MSP1 or PvMSP1_19_ in mice were determined by ELISA using Immulon 2HB plates (Thermo Scientific, Waltham, MA) coated with 1 μg/ml of PvRMC-MSP1 or PvMSP1_19_ diluted in carbonate buffer, or peptides representing the *P. vivax* promiscuous T cell epitopes in PBS as described^9^. Optical densities were determined using a 405 nm filter on a VERSAmax ELISA reader (Molecular Device Corporation, Sunnyvale, CA). The cutoff value was set at the highest dilution of sera resulting in an O.D. greater than three standard deviations (S.D.) above the mean obtained using sera from unimmunized mice. ELISA results are presented as the reciprocal of the end-point dilution.

IgG subclass profiles were determined by ELISA. Following incubation with mouse sera, plates were washed and incubated for 90 minutes with biotinylated anti-mouse IgG1 or IgG2a rat mAbs (BD PharMingen, Franklin Lakes, NJ). Plates were washed again, and the bound antibodies were detected using horseradish peroxidase (HRP)-streptavidin (BD PharMingen) and H_2_O_2_/2,2-azinobis (3-ethylbenzthiazoline-6-sulfonic acid) (ABTS) as substrate (KPL).

The avidity of anti-PvRMC-MSP1 antibodies was assessed by a thiocyanate elution-based ELISA using sera samples obtained 20 days after the third immunization (day 60). The assay was conducted in the same manner as described above with a slight modification^24^. In brief, 0–10 M ammonium thiocyanate (NH_4_SCN) in PBS was added to each well after incubation with the sera dilutions. The plates were incubated at room temperature for 15 min and then washed before the addition of the secondary anti-mouse IgG antibody. Serial dilutions of the sera were assayed in the presence or absence of 1 M NH_4_SCN to determine avidity index. For these experiments, the washing step before incubation with anti-mouse IgG antibody was duplicated to remove weakly bound IgG antibodies. The logarithms of reciprocal serum dilutions corresponding to the half-maximum absorbance value in curves obtained with and without NH_4_SCN, termed x_1_ and x_2_, were interpolated by third-degree polynomial regression. The avidity index is the ratio between the antilog of x_1_ and the antilog of x_2_, or simply antilog (x_1_−x_2_) as described by Ferreira and Katzin^92^.

Absorption ELISA. To test if conformational epitopes are preserved in PvRMC-MSP1, polyclonal antibodies obtained from mice immunized with PvMSP1_19_ were tested for reactivity against reduced or non-reduced PvRMC-MSP1 by ELISA. To remove antibodies elicited against linear epitopes the samples tested in ELISA were first absorbed with reduced PvRMC-MSP1 at 2 μg/ml for 2 hours. For absorption under reducing conditions, PvRMC-MSP1 was initially treated with 0.05 M dithiothreitol (DTT) at 37 °C for 1 h. The reduced PvRMC-MSP1 was then diluted in 0.1 M carbonate buffer, containing 0.05 M DTT as described^93^ and Immulon 2HB plates coated overnight. After 2 hours absorption, the samples were then tested for recognition of reduced or non-reduced PvRMC-MSP1 at 1 μg/ml. To maintain the reducing conditions during the ELISA tests and avoid unspecific antibody binding mediated by the use of DTT, the washing buffer, blocking buffer and solvent buffers included 5 mM EDTA. Results are presented as a curve of mean absorbance values of four technical replicates versus antibody concentration.

### Indirect immunofluorescence assays

Sera obtained from BALB/c and C57BL/6 mice after the third immunization with 20 μg of the PvRMC-MSP1 were pooled, and the antibody reactivity against native protein was evaluated using indirect immunofluorescence assays. Blood was collected from a *P. vivax* infected *Saimiri boliviensis* monkeys (aliquot kindly provided by Dr. Mary Galinski) or a *P. coatneyi* infected rhesus macaques into CPD tubes. The blood samples were washed twice using RPMI 1640 medium and the cells adjusted to 1% hematocrit. To each well of a 12-well slide (ICN Biomedicals Inc., Aurora, OH) 10 μl of the cell suspension was added and slides air-dried before being stored at −20 °C. Following storage at −20 °C, parasites slides were air dried at room temperature and subsequently incubated with the different dilutions of mouse sera obtained after the third immunization diluted in PBS+0.2% BSA in a dark, moist chamber for 90 min. After the incubation, slides were washed three times with PBS containing Tween-20 (PBST), to minimize non-specific binding. Parasites were stained for 30 min at room temperature in a dark, moist chamber with goat anti-mice Alexa Fluor 488 (Invitrogen Corporation, Carlsbad, CA) at a 1:500 dilution in Evans Blue 0.4%. After staining, slides were washed three times, and parasite nuclei were visualized using 4′,6-diamidino-2-phenylinodole dihydrochloride (DAPI) included in the anti-fade mounting medium Prolong (Life Technologies, Grand Island, NY).

### Flow cytometry assays

Multiparametric flow cytometry analysis of PvRMC-MSP1-specific T cells was conducted using an eight-color panel which allowed for simultaneous analysis of IL-2, IFN-γ, and TNF-α at the single-cell level in T cells derived from splenocytes obtained five days after the final boosting immunization. A library of 61 synthetic peptides representing the complete amino acid sequence of the chimeric protein without carboxyl terminal tags was used for *ex vivo* stimulation (Supplementary Table S3). Cells were stimulated for 6 hours with peptide pools at a concentration of 2 μg/ml per peptide or with PvRMC-MSP1 or PvMSP1_19_ at 2 μg/ml at 37 °C in the presence of GolgiPlug (BD Biosciences, San Jose, CA). Cells were then incubated with Live/Dead stain at 430 nm excitation (Life Technologies) followed by surface staining with α-CD3 (PerCP Cy5.5), α-CD4 (Alexa Fluor 700), and α-CD8α (APC-Cy7) for 30 min. The cells were then fixed, permeabilized, and stained with antibodies against IFN-γ (FITC), TNF-α (PE), and IL-2 (APC). All the monoclonal antibodies were obtained from BioLegend (San Diego, CA). Flow cytometry analyses were performed using an LSRII flow cytometer (BD Biosciences, San Jose, CA). Data were analyzed using FlowJo V10.1 software, beginning with the selection of single cell and then the lymphocytes gated in SSC-A vs. FSC-A. Cells were then gated on the Live/Dead channel, and then CD3 + CD4+ and CD3 + CD8+ antigen-specific cytokine-secreting T cells were identified (Supplementary Figure S7). The frequency of antigen-specific cytokine-producing cells was determined by subtracting the percentage of cytokine-producing T cells after incubation with medium alone from the percentage of cytokine-producing T cells after incubation with PvRMC-MSP1, the PvMSP1_19_ protein, or the corresponding peptide pools. A threshold for a positive cytokine response was set above the background (sample incubated with medium alone), and samples that did not meet this requirement were set to zero.

### Seroprevalence study area and volunteers

The characteristics of the study participants have been previously described^27^,^35^. Briefly, 253 individuals from the malaria endemic region of Rondonia, Brazil were included in this survey. *P. vivax* accounts for more than 70% of all malaria cases in this region^35^,^94^. During June through August (the period of increased malaria transmission) of 2004 (n = 202) and 2007 (n = 56) samples and survey data were collected. Most of the population used for this study consist of rain forest natives who have resided for over 25 years in the malaria-endemic region or transmigrants originating from several non-endemic regions that have lived in Rondonia for more than ten years. For controls, samples from 30 individuals from Rio de Janeiro, a malaria non-endemic area, with no history of malaria or residence in endemic areas were used. The study was performed in accordance with the principles of the Declaration of Helsinki and in accordance with Good Clinical Practice (GCP). Review and approval of all the experimental protocols was granted by the Oswaldo Cruz Foundation Ethical Committee IRB No. 138/01 and 354/06 and the National Ethical Committee of Brazil.

### Epidemiological survey

All the individuals who agreed to participate in our study signed an informed consent document formalizing their participation as volunteers. Volunteers were interviewed to record epidemiological data. Survey data was entered into a database created with Epi Info 2002 (Centers for Disease Control and Prevention, Atlanta, GA).

### Human blood samples and malaria diagnosis

Heparinized tubes were used to obtain plasma from 10 ml whole blood samples. The plasma was separated from all blood samples and stored at −20 °C before being shipped on dry ice to the Immunoparasitology Laboratory, IOC, Fiocruz. Diagnosis of malaria was made using thin and thick blood smears, stained with Wright-Giemsa stain (Sigma Chemical Co., St. Louis, USA). Parasitemia was determined for donors positive for malaria as the frequency of parasites (all species and stages present) per 200 leukocytes in the thick smear. Of the positive donors, six were positive for *P. falciparum* and eighteen were positive for *P. vivax*. These individuals were subsequently treated for their infection per the regimen recommended by the Brazilian Ministry of Health.

### Human Antibodies Determination

The presence of antibodies against PvRMC-MSP1 in the plasma of the volunteers was assessed using ELISA as previously described^27^,^35^. Briefly, 200 ng of PvRMC-MSP1 was used to coat 96-well Maxisorp plates (Nunc, Rochester, NY). Plates were incubated overnight at 4 °C and washed with a 0.05% Tween 20 solution in PBS (PBS-Tween). Plates were then blocked for 1 hour at 37 °C using 5% non-fat dry milk diluted in PBS-Tween (PBS-Tween-M). Plasma samples were diluted individually at 1:100 in PBS-Tween-M. Plasma samples were then added to the plates in duplicate and incubated for 1 hour at 37 °C. Following incubation, plates were washed four times with PBS-Tween and incubated with peroxidase-conjugated goat anti-human IgG (Sigma, St. Louis, MO) at 1:1000 dilution. O-phenylenediamine and hydrogen peroxide were used to reveal bound antibodies. The absorbance was read on Spectramax 250 ELISA reader (Molecular Devices, Sunnyvale, CA) using a 492 nm filter. The results for total IgG were expressed as reactivity indexes (RI) that were calculated as the mean optical density of tested samples divided by the mean optical density plus 3 standard deviations of 5 non-exposed controls tested on each plate. Subjects were scored positive for serum IgG to a particular antigen if the RI was higher than 1.

In addition, IgG subclasses were determined in individual responders by ELISA. The following peroxidase conjugated monoclonal mouse anti-human antibodies were used: mouse anti-human IgG1 (hinge)-HRP (clone HP6001, Southern Biotechnology); mouse anti-human IgG2 (Fc)-HRP (clone HP6002, Southern Biotechnology); mouse anti-human IgG3 (hinge)-HRP (clone HP6050, Southern Biotechnology) and mouse anti-human IgG4 (Fc)-HRP (clone HP6023, Southern Biotechnology). All antibodies were diluted by 1:1000. Samples were considered subclass-specific positive for each antigen when the OD values were 3 standard deviations above the mean OD of four non-exposed controls.

### Absorption treatment ELISA

To ensure that the naturally acquired antibodies detected in ELISA were directed to PvRMC-MSP1 and not to the (NANP)_6_ tag used for biochemical characterization, we performed an IgG absorption ELISA protocol using a synthetic (NANP)_6_ peptide. Briefly, flat-bottom plates (NUNC, USA) were coated overnight with 5 μg/mL of the peptide (NANP)_6_. After washing and blocking steps, plasma from 63 randomly selected PvRMC-MSP1 IgG responders were added to the plates at a 1:100 dilution and incubated for two hours. After incubation, plasma samples were transferred to plates coated with PvRMC-MSP1 (200 ng) and the ELISA was performed as previously described.

### HLA Genotyping of PBMCs

Whole blood samples drawn in EDTA were used to isolate genomic DNA by incubating samples for 1 hour at 50 °C with a mixture of 95 μl of proteinase K (20 mg/ml) and 5 ml buffer G2 (QIAamp DNA Blood Midi Kit; Qiagen Inc., Chatsworth, CA, USA). Following incubation, DNA was ethanol precipitated and collected with a glass rod and transferred into distilled water. The concentration and quality of DNA were assessed using a NanoDrop ND-1000 spectrometer (Thermo Fisher Scientific Inc., Waltham, MA, USA). Luminex xMAP technology and sequence-specific oligonucleotide probes (SSOPs) were used to determine HLA-DQB1 and HLA-DRB1 allelic groups within the study population. Briefly, this system is based on the use of probe arrays which are bound to color-coded plastic microspheres and locus specific biotinylated primers for HLA-DQB1 and HLA-DRB1 loci (LABType, One Lambda Inc, Canoga Park, CA, USA). Biotinylated amplicons were denatured to ssDNA and incubated with DNA complementary probes immobilized on fluorescently coated microspheres (beads). Samples were then incubated with R-phycoerythrin-conjugated streptavidin. After hybridization, the samples were analyzed using a Luminex Flow Analyzer. The HLA Visual 2.0 software (One Lambda, CA) analysis program deduces the HLA-DQB1 and HLA-DRB1 allelic groups.

### Statistical Analysis

Statistical analysis and graphs were made using GraphPad Prism 5.0 software (GraphPad Software Inc., San Diego, CA). For analysis of the mouse immunogenicity studies, all ELISA titers were log-transformed to conform to the normality, and variance requirements of parametric testing and groups were compared using Student’s t-test or one-way ANOVA with post hoc Bonferroni’s multiple comparison post-test when suitable. Cellular responses were analyzed with Mann-Whitney tests for comparison of non-normally distributed data.

For human data, analyses were done as previously described^35^ using Epi Info 2002 (CDC, Atlanta, GA), and GraphPad Prism 5.0 (GraphPad Software, San Diego, CA) according to the required statistical test. Differences in medians for the study population data were tested by non-parametric Mann–Whitney test when appropriate. Student’s t-test was used to compare the means of normally distributed data or normalized transformations were performed on raw data before testing by one-way ANOVA where appropriate. Differences in the proportions of the frequencies between variables were evaluated by chi-square (χ^2^) test. Relationships between the RI against PvRMC-MSP1 and age, years of residence in the endemic area, time of residence in Rondonia, the number of past malaria episodes or months since last known malaria episode were assessed with Spearman’s rank correlation. Allelic groups were grouped by DR status, and data were descriptively summarized using frequencies and percentages for all categorical variables. Overall associations of immunological responses with the alleles from each HLA-DRB1* and HLADQB1* loci were evaluated by comparing the allele frequencies between seronegative subjects and seropositive subjects using standard contingency tables. Each person contributed two observations to the table (one for each allele). Rare alleles, defined as those with less than five occurrences among subjects, were all pooled into a category labeled “other” for analysis. To evaluate global differences in allele distribution, we performed analyses using simulation methods as implemented in the software PASW. This approach randomly generates new cell counts for contingency tables under the null hypothesis of no association, while keeping the margins of the table fixed. We used an approach that compares each allele versus all others combined, resulting in multiple 262 tables, and used the maximum χ^2^ statistic from this series of tables as a global test statistic (bipartition). All statistical tests were two-sided, and HLA analyses were conducted using the PASW software system.

## Additional Information

**How to cite this article**: Fonseca, J. A. *et al*. A chimeric protein-based malaria vaccine candidate induces robust T cell responses against *Plasmodium vivax* MSP1_19_. *Sci. Rep.* **6**, 34527; doi: 10.1038/srep34527 (2016).

## Supplementary Material

Supplementary Information

## Figures and Tables

**Table 1:** Amino acid sequence homology between *Plasmodium vivax* promiscuous T cell epitopes included in PvRMC-MSP1 and simian *Plasmodium* Species.

Promiscuous T cell Epitope	*Plasmodium vivax*^a^ (GenBank XM_001614792.1)	*Plasmodium coatneyi*^a^,^b^ (GenBank BAF74048)	Homology (%)	*Plasmodium cynomolgi*^a^,^b^ (GenBank BAI82251)	Homology (%)
PvT4	NFVGKFLELQIPGHTDLLHL	**D**FVGK**Y**LELQIPGH**AN**LLH**M**	75	**D**FVGKFLELQIPGHT**N**LLH**M**	85
PvT6	FNQLMHVINFHYDLLRANVH	FNQLMHV**V**NF**N**YDLLRA**KLN**	70	FNQLMHVINFHYDLLRA**KLN**	85
PvT8	LDMLKKVVLGLWKPLDNIKD	LDMLKKVVLG**YR**KPLDNIKD	90	LDMLKKVVLG**YR**KPLDNIKD	90
PvT19	LEYYLREKAKMAGTLIIPES	LEYYLREKAKMAGTLI**A**PES	95	LEYYLREKAKMAGTLI**T**PES	95
PvT53	SKDQIKKLTSLKNKLERRQN	SK**EH**IKKLTSLKNKLERRQN	90	SK**E**QIKKLTSLKNKLERRQN	95

^a^Underline Denotes P1-P6 motif.

^b^Bold denotes amino acid changes from the *P. vivax* sequence.

**Table 2:** Anti-PvRMC-MSP1 antibody prevalence in Brazilian donors.

Epidemiological Factor	Relation with Anti-PvRMC-MSP1 Antibodies	
	Spearman (r)	p value
Age	0.1112	0.07
Time of residence in malaria endemic area	−0.038	0.53
Time of residence in Rondonia	0.0007	0.99
Number of past malaria infections	**0.1795**	**0.003**
Number of months since the last infection	**−0.3101**	**<0.0001**
