# Supplementary material for: A chimeric protein-based malaria vaccine candidate induces robust T cell responses against Plasmodium vivax MSP119
Source: Sci Rep. 2016 Oct 6;6:34527. doi: 10.1038/srep34527 (PMC5052570; doi:10.1038/srep34527)
Supplement: Supplementary Information [file srep34527-s1.pdf]

Supplementary Information:

A chimeric protein-based malaria vaccine candidate induces robust T cell responses against *Plasmodium vivax* $MSP1_{19}$.

Jairo Andres Fonseca[1,2], Monica Cabrera-Mora[1], Balwan Singh[1], Joseli Oliveira-Ferreira[3], Josué da Costa Lima-Junior[3], J. Mauricio Calvo-Calle[4], Jose Manuel Lozano[5], Alberto Moreno[1,2,*].

Road, Atlanta, GA 30329.

2. Division of Infectious Diseases, Department of Medicine, Emory University, 69 Jesse Hill, Jr. Drive, SE, Atlanta, GA 30303.

3. Laboratory of Immunoparasitology, Oswaldo Cruz Institute, Oswaldo Cruz Foundation, (FIOCRUZ), Rio de Janeiro, RJ, Brazil.

4. Department of Pathology, University of Massachusetts Medical School, Worcester, MA.

5. Molecular Mimetism of Infectious Agents Unit, Pharmacy Department, Universidad Nacional de Colombia, Bogota D.C., Colombia.

*Corresponding Author: Emory Vaccine Center, Yerkes National Primate Research Center, Emory University. 954 Gatewood Road. Atlanta, GA 30329. Fax: (404) 727-8199. Phone: (404) 727-8611. E-mail: alberto.moreno@emory.edu

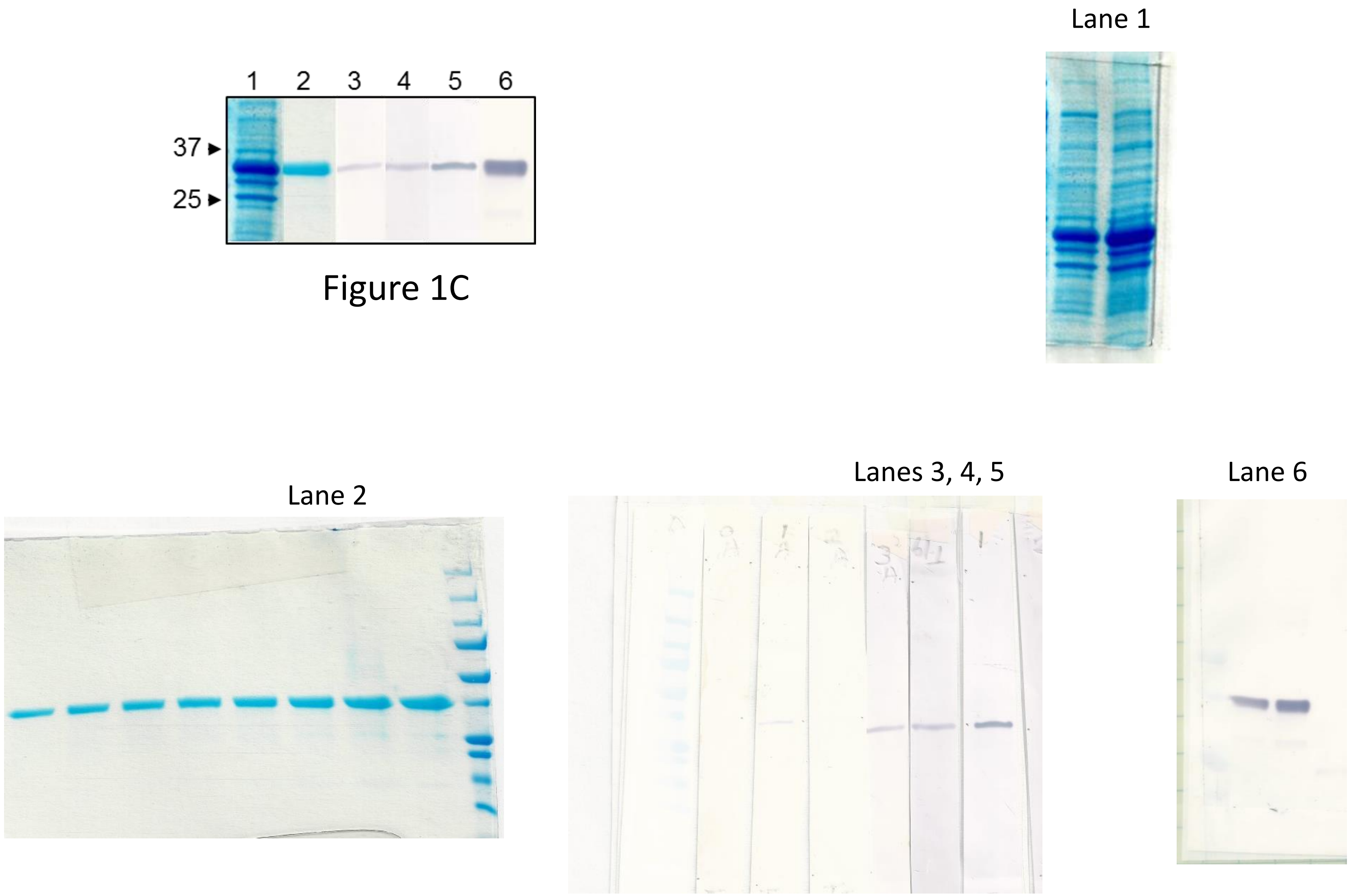

Supplementary Figure S1. Full unedited gels/western blots for Figure 1C. The Figure 1C and the gels/Western Blots that compose the figure are presented. **Upper right gel.** Coomassie stain after SDS-PAGE separation of *E. coli* lysate after induction with IPTG (Lane 1 on Figure 1C)**. Bottom left gel.** Coomassie stain after SDS-PAGE separation of the purified PvRMC-MSP1 separated on a 4-20% gradient gel (Lane 1 and 2 on Figure 1C). **Bottom left western blot**. PvRMC-MSP1 incubated with sera samples from mice immunized with a synthetic peptide representing the T cell epitope L_{158}-D_{177} (Lane 3 Figure 1C), a synthetic peptide representing the T cell epitope L_{378}-S_{397} (Lane 4 Figure 1C), and a synthetic peptide representing the T cell epitope N_{78}-L_{97} (Lane 5 Figure 1C). **Bottom right western blot.** Incubation of PvRMC-MSP1 with the monoclonal antibody 2A10 that recognizes the $(NANP)_6$ repeat sequence used as a C-terminal tag (Lane 6 Figure 1C).

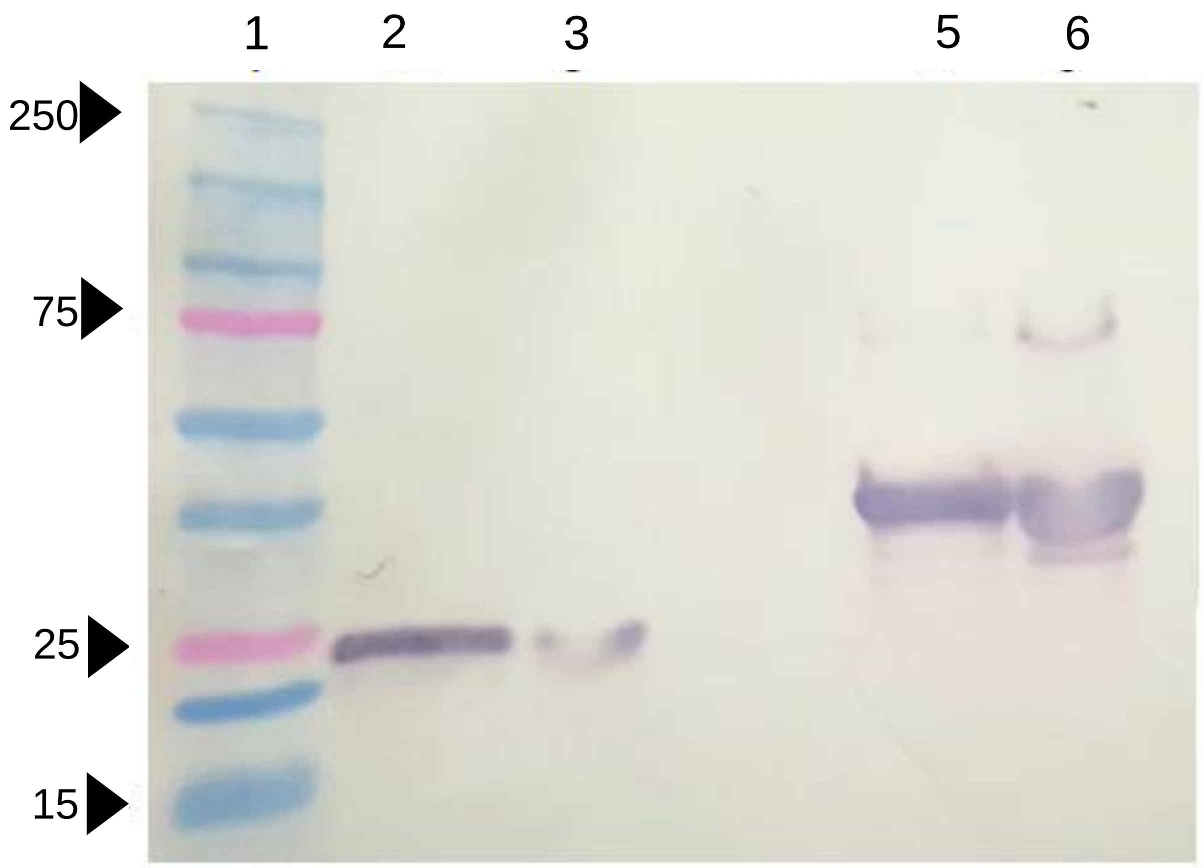

Supplementary Figure S2. Western blot analyses of PvRMC-MSP1 and PvMSP1$_{19}$ after SDS-PAGE in reducing and non-reducing conditions. Both PvMSP1$_{19}$ (Lanes 2 and 3) and PvRMC-MSP1 (Lanes 5 and 6) were resolved using 5-20% gradient SDS-PAGE and then subject to western blot analysis with α-His tag monoclonal antibody. Reduced conditions are presented on Lanes 3 and 6. The molecular weight markers (Lane 1) are indicated.

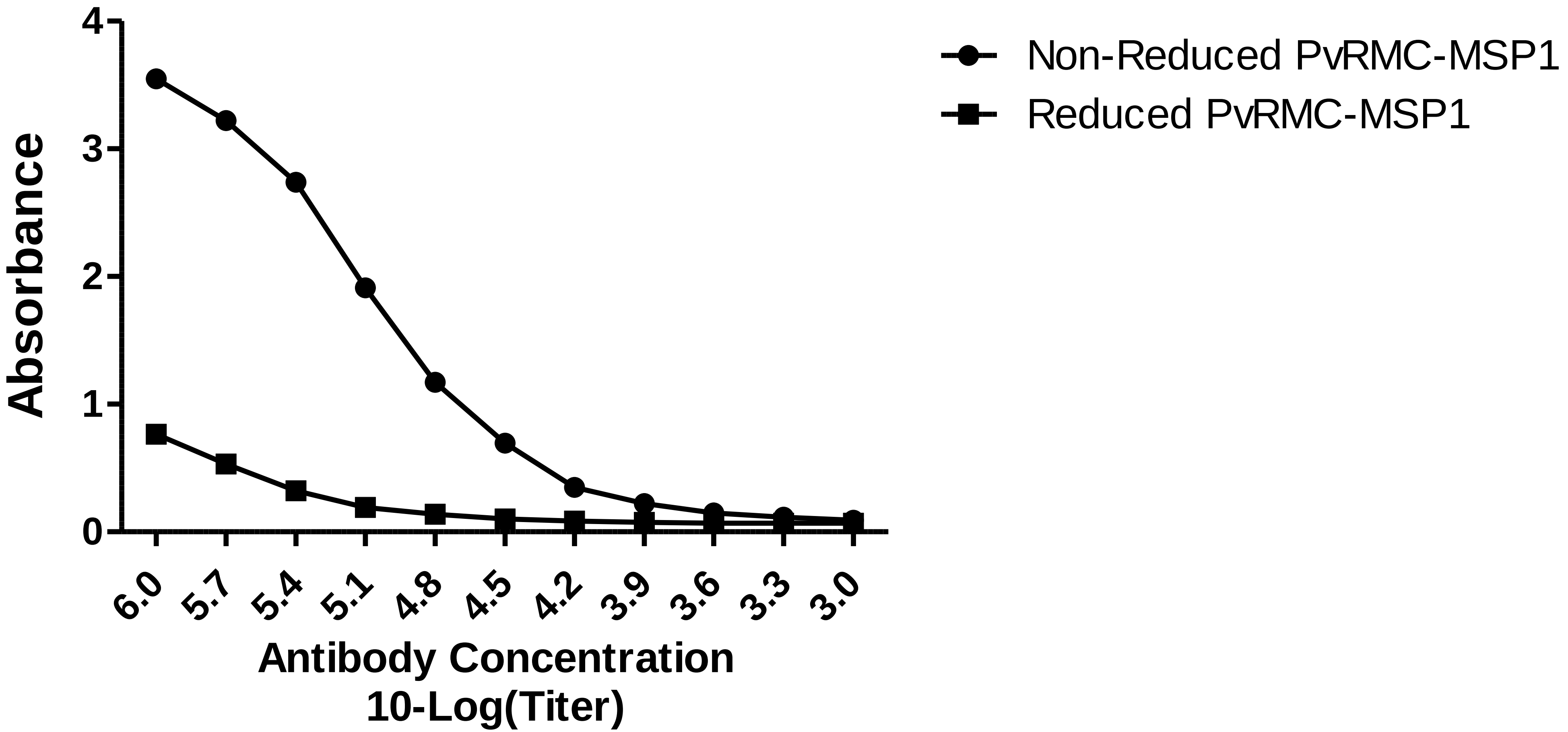

Supplementary Figure S3. PvMSP1$_{19}$ conformational antibodies against PvRMC-MSP1 in reduced and non-reduced conditions. Polyclonal antibodies from mice immunized with PvMSP1$_{19}$ were incubated for 2 hours with 2 µg/ml of reduced PvRMC-MSP1 to absorb linear PvMSP1$_{19}$ antibodies as described in the Methods section. After absorption, the conformational antibodies were tested against reduced and non-reduced PvRMC-MSP1 proteins at 1µg/ml using ELISA. Results are presented as a curve of absorbance vs. antibody concentration. Each point is the average of four technical replicates.

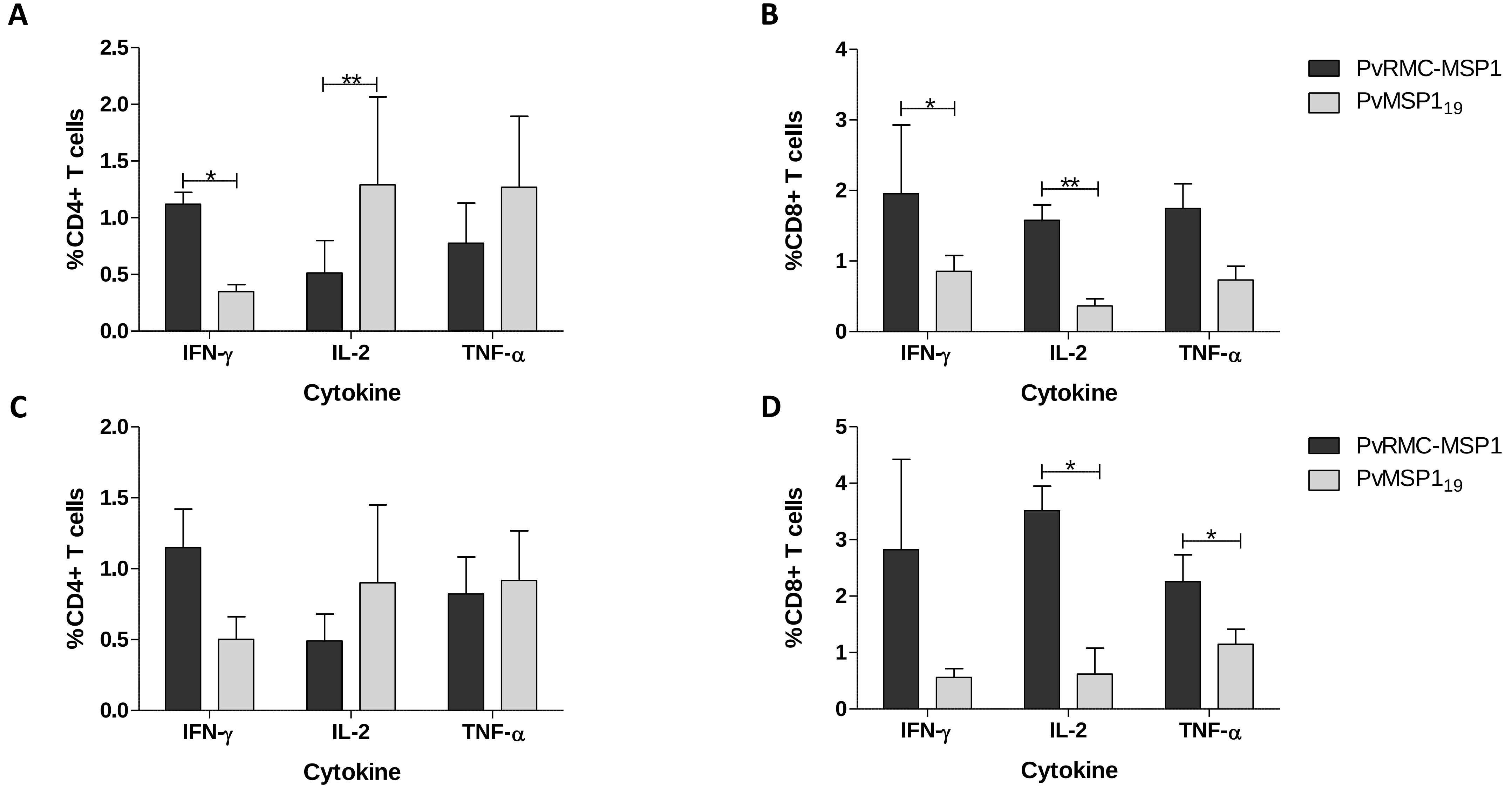

Supplementary Figure S4. T cell responses to the different components of PvRMC-MSP1 in C57BL/6 mice. Five days after the final immunization spleens from mice immunized with either PvRMC-MSP1 (n=5) or PvMSP1$_{19}$ (n=5) were processed and stimulated with peptide pools representing the different components of PvRMC-MSP1 and cytokine-secreting T cells assessed. Top Panel. CD4+ **(A)** and CD8+ **(B)** cytokine secreting T cells in C57BL/6 mice after stimulation with peptide pools representing the promiscuous T cell epitopes present in PvRMC-MSP1. Bottom Panel. CD4+ **(C)** and CD8+ **(D)** secreting T cells in C57BL/6 mice after stimulation with peptide pools representing the PvMSP1$_{19}$ molecule present in PvRMC-MSP1. Results are presented after background subtraction. Statistical analysis was done using Mann-Whitney test. *p<0.05.

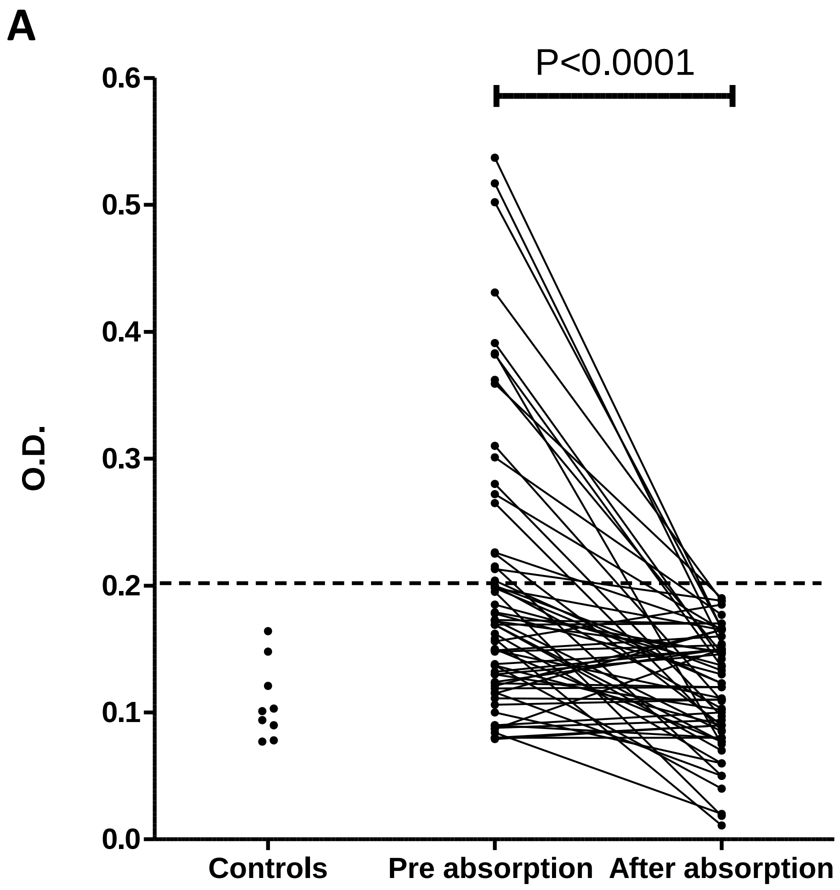

Supplementary Figure S5. **Antibody recognition of $(NANP)_6$ by individuals naturally exposed to malaria.** Optical densities **(A)** and Reactivity Index **(B)** of the anti-NANP antibodies of the 63 randomly selected individuals from the studied population are presented. After absorption of the anti-NANP antibodies, a significant reduction on both the O.D. and RI in the anti-NANP ELISA was observed after analysis with the Wilcoxon matched-pairs signed rank test.

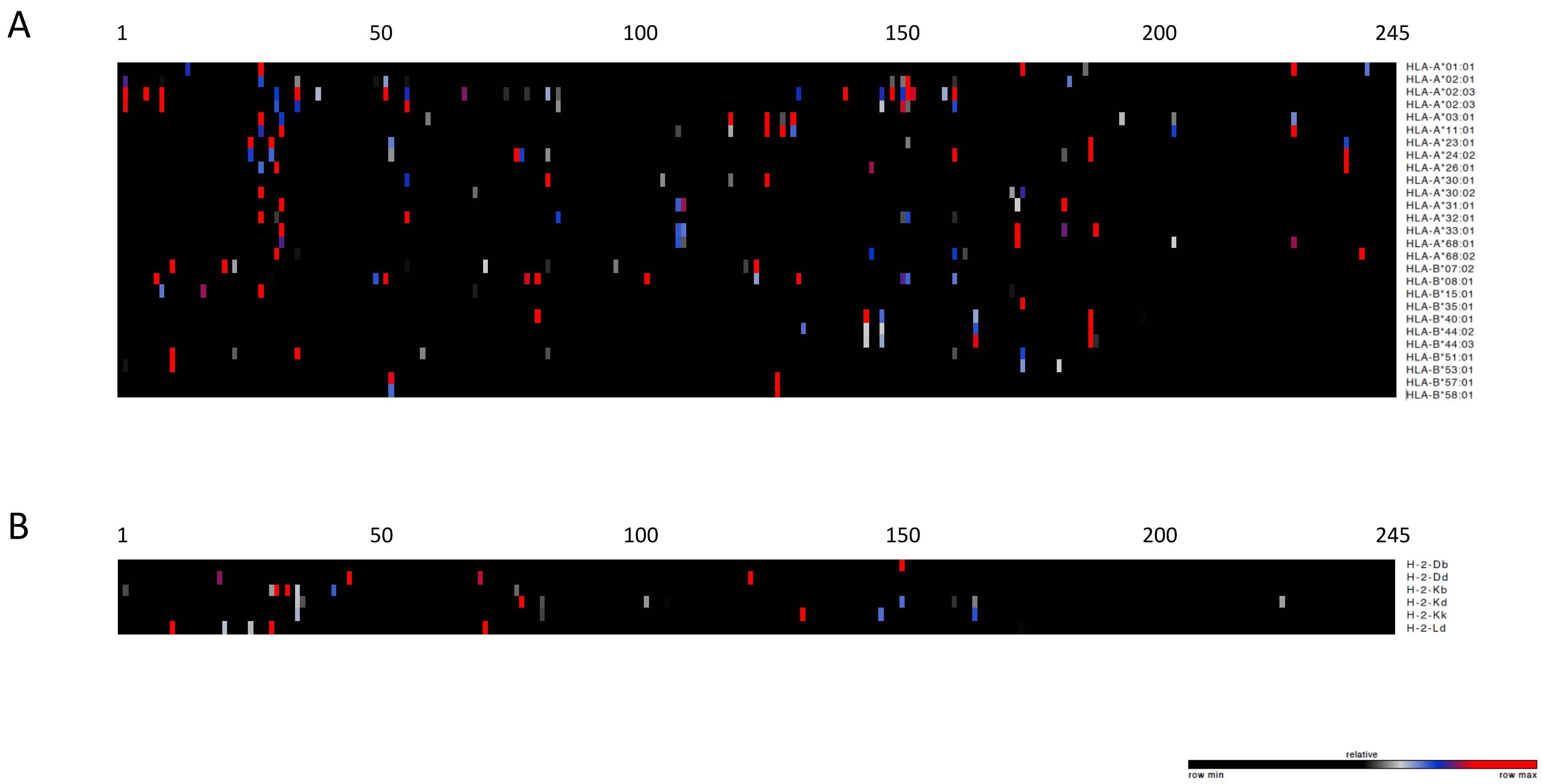

Supplementary Figure S6. Predicted HLA class I binding peptides in PvRMC-MSP1. Data are shown as heat maps generated from predicted scores by the IEDB server for binding to human (A) and mouse (B) MHC class I alleles. Columns indicate amino acid position in PvRMC-MSP1 and in the right, the MHC class I allele are indicated for which the prediction was performed. Peptides predicted to have high affinities (<50nM) in red; medium affinity (>50 to 500 nM) in blue, low affinity (<500 to 5000 nM) in gray and very low to no binding in black (>5000nm). PvRMC-MSP1 is predicted to contain several peptides with medium to high affinity for binding to HLA-A* alleles in the N-terminal and central region. Peptides predicted to have high to medium affinities for HLA-B* are less numerous. Binding of PvRMC-MSP1 peptides to mouse class I alleles is predicted to be more restricted and observed mainly in the first 100 amino acids. Heat maps were generated by using the matrix visualization and analysis platform GENE-E (http://www.broadinstitute.org/cancer/software/GENE-E/index.html). The MHCI binding predictions were made on 12-14-15 using the IEDB analysis resource ANN tool [59,63,66,67]

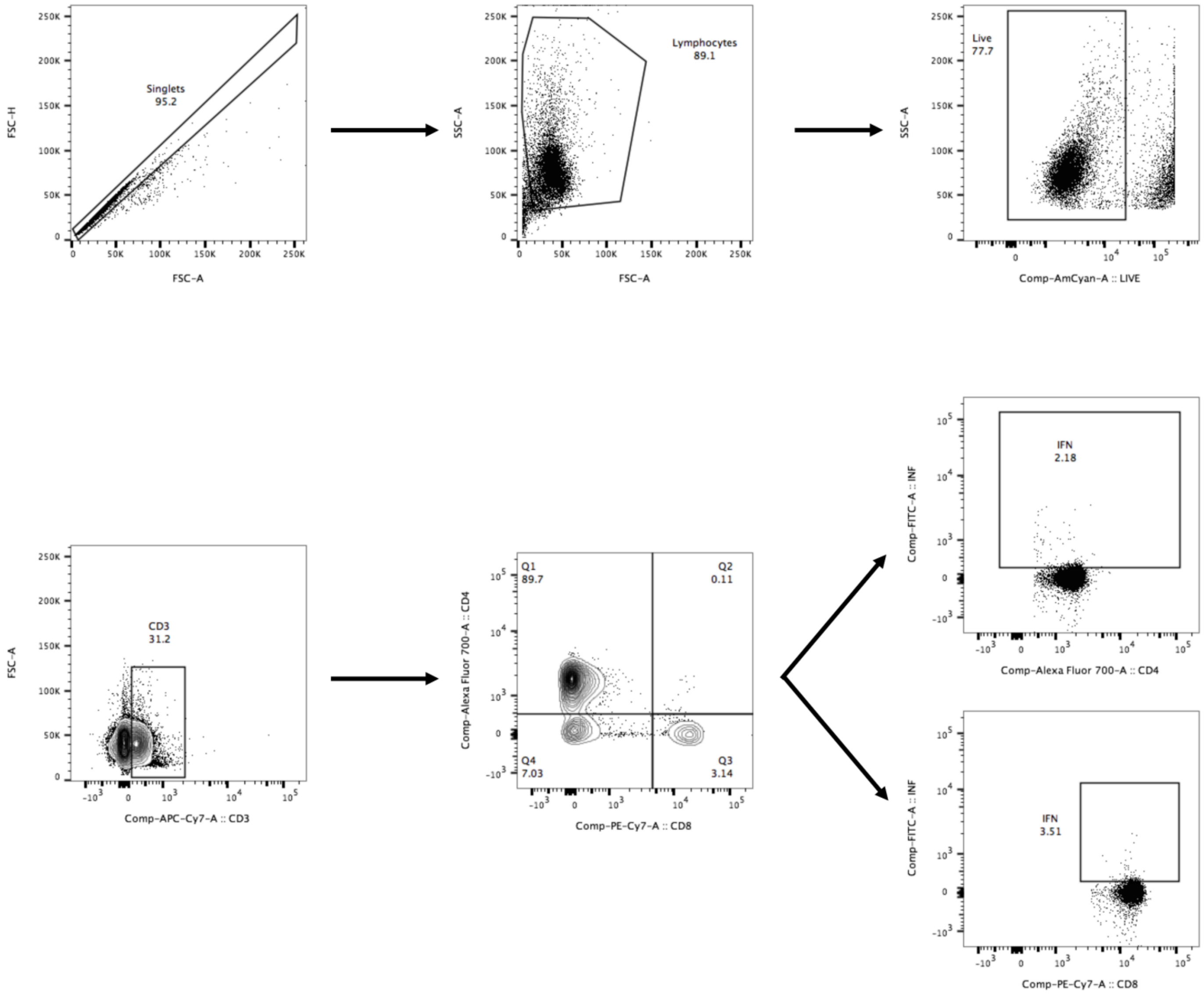

Supplementary Figure S7. Gating Strategy For Flow Cytometry Analysis. In this sample, gating cells were first gated for singlets (FSC-H vs. FSC-A) and lymphocytes (SSC-A vs. FSC-A). The lymphocyte gate is further analyzed for their uptake of the Live/Dead stain to determine live versus dead cells. After selection of the live population, the CD3 expression was then analyzed to select the T cell population. CD4 and CD8 surface expression were then determined. Intracellular expression of cytokines (IFN-γ, TNF-α, IL-2) was analyzed in each T cell subset.

Supplementary Table S1. Homologies between *P. vivax* MSP-1 and NHP *Plasmodium* species MSP-1

	MSP-1_{33} T epitope
P. vivax (GenBank XM_001614792.1)	VKSSGLLEKLMKSKLIKENESKEILSQLLNVQTQLL
P. coatneyi (GenBank BAF74048)	VKSSGLLEKFKNSKLINEEESKKVLSQLLNMQTKML
Homology %	75.0
P. cynomolgi (GenBank BAI82251)	VKSSGLLEKLMNSKLINQEESKKALSELLNVQTQML
Homology %	77.8
	**MSP-1_{19} Fragment**
P. vivax (GenBank XM_001614792.1)	TMSSEHTCIDTNVPDNAACYRYLDGTEEWRCLLTFKEEGGKCVPASNVTCKDNNGGCAPEAECKMTDSNKIVCKCTKEGSEPLFEGVFCSSSS
P. coatneyi (GenBank BAF74048)	NMGSEHKCIDTTVPENAACYRYLDGTEEWRCLLNFKELEGKCIPAPDMTCNENNGGCAPEAECKMTESKKIVCKCTKEGSEPLFDGVFCSSSS
Homology %	82.8
P. cynomolgi (GenBank BAI82251)	NMSSEHRCIDTNVPENAACYRYLDGTEEWRCLLYFKEDAGKCVPAPNMTCKDKNGGCAPEAECKMNDKNEIVCKCTKEGSEPLFEGVFCSHHH
Homology %	83.9

Supplementary Table S2. Promiscuous T Cell Epitopes Homology Between *P. vivax* Strains

Strain	PvT4[a,b]	PvT6	PvT8	PvT19	PvT53
P. vivax Belem<br>(GenBank M60807)	NFVGKFLELQIPGHTDLLHL	FNQLMHVINFHYDLLRANVH	LDMLKKVVLGLWKPLDNIKD	LEYYLREKAKMAGTLIIPES	SKDQIKKLTSLKNKLERRQN
P. vivax North Korea<br>(GenBank KNA00298.1)	NFV**S**KFLELQIPGHTDLLHL	FNQLMHVINFHYDLLRA**KL**H	LDMLKKVVLG**YR**KPLDNIKD	LEYYLREKAKMAGTLI**V**PES	SKDQIKKLTSLKNKLERRQN
Homology %	95.0	90.0	90.0	95.0	100.0
P. vivax India VII<br>(GenBank KMZ81136.1)	NFVGKFLELQIPGHTDLLHL	FNQLMHVINFHYDLLRA**KL**H	LDMLKKVVLG**YR**KPLDNIKD	LEYYLREKAKMAGTLIIPES	SKDQIKKLTSLKNKLERRQN
Homology %	100.0	90.0	90.0	100.0	100.0
P. vivax Mauritania I<br>(GenBank KMZ93811.1)	NFV**S**KFLELQIPGHTDLLHL	FNQLMHVINFHYDLLRA**KL**H	LDMLKKVVLG**YR**KPLDNIKD	LEYYLREKAKMAGTLIIPES	SKDQIKKLTSLKNKLERRQN
Homology %	95.0	90.0	90.0	100.0	100.0
P. vivax Brazil I<br>(UniProt KB A0A0J9SVZ4)	NFV**S**KFLELQIPGHTDLLHL	FNQLMHVINFHYDLLRA**KL**H	LDMLKKVVLG**YR**KPLDNIKD	LEYYLREKAKMAGTLI**V**PES	SKDQIKKLTSLKNKLERRQN
Homology %	95.0	90.0	90.0	95.0	100.0
P. vivax Salvador I<br>(UniProt KB A5K724)	NF**LS**KFLELQIPGHTDLLHL	FNQLMHVINFHYDLLRA**KL**H	LDMLKKVVLG**YR**KPLDNIKD	LEYYLREKAKMAGTLIIPES	SKDQIKKLTSLKNKLERRQN
Homology %	90.0	90.0	90.0	100.0	100.0

[a]Underline Denotes P1-P6 motif.

[b]Bold red characters denote amino acid changes from the *P. vivax* Belem sequence.

Supplementary Table S3. Peptides pools used for Flow Cytometry	
Peptide Used	Sequence
MSP-1 Promiscuous T cell Epitopes	MANFVGKFLELQIPG<br>VGKFLELQIPGHTDL<br>LELQIPGHTDLLHLG<br>IPGHTDLLHLGPGPG<br>TDLLHLGPGPGFNQL<br>HLGPGPGFNQLMHVI<br>GPGFNQLMHVINFHY<br>NQLMHVINFHYDLLR<br>HVINFHYDLLRANVH<br>FHYDLLRANVHGPGP<br>LLRANVHGPGPGLDM<br>NVHGPGPGLDMLKKV<br>PGPGLDMLKKVVLGL<br>LDMLKKVVLGLWKPL<br>KKVVLGLWKPLDNIK<br>LGLWKPLDNIKDGPG<br>KPLDNIKDGPGPGLE<br>NIKDGPGPGLEYYLR<br>GPGPGLEYYLREKAK<br>GLEYYLREKAKMAGT<br>YLREKAKMAGTLIIP<br>KAKMAGTLIIPESGP<br>AGTLIIPESGPGPGS<br>IIPESGPGPGSKDQI<br>SGPGPGSKDQIKKLT<br>PGSKDQIKKLTSLKN<br>DQIKKLTSLKNKLER<br>KLTSLKNKLERRQNG<br>LKNKLERRQNGPGPG<br>LERRQNGPGPGVKSS
MSP-1_{33} T cell peptides and MSP-1_{19}	QNGPGPGVKSSGLLE<br>GPGVKSSGLLEKLMK<br>KSSGLLEKLMKSKLI<br>LLEKLMKSKLIKENE<br>LMKSKLIKENESKEI<br>KLIKENESKEILSQL<br>ENESKEILSQLLNVQ<br>KEILSQLLNVQTQLL<br>SQLLNVQTQLLTMSS<br>NVQTQLLTMSSEHTC<br>QLLTMSSEHTCIDTN<br>MSSEHTCIDTNVPDN<br>HTCIDTNVPDNAACY<br>DTNVPDNAACYRYLD<br>PDNAACYRYLDGTEE<br>ACYRYLDGTEEWRCL<br>YLDGTEEWRCLLTFK<br>TEEWRCLLTFKEEGG<br>RCLLTFKEEGGKCVP<br>TFKEEGGKCVPASNV<br>EGGKCVPASNVTCKD<br>CVPASNVTCKDNNGG<br>SNVTCKDNNGGCAPE<br>CKDNNGGCAPEAECK<br>NGGCAPEAECKMTDS<br>APEAECKMTDSNEIV<br>ECKMTDSNEIVCKCT<br>TDSNEIVCKCTKEGS<br>EIVCKCTKEGSEPLF<br>KCTKEGSEPLFEGVF<br>EGSEPLFEGVFCSSS

Supplementary Table S4. Frecuency and HLA association with anti-PvRMC-MSP1 responses

HLA Allele	PvRMC-MSP1	
	Responder	Non-responder
HLA-DRB1*	F	F
DRB1*01	0,217	0,187
DRB1*03	0,155	0,117
DRB1*04	0,31	0,328
DRB1*07	0,209	0,179
DRB1*08	0,178	0,187
DRB1*09	0,023	0,039
DRB1*10	0,023	0,023
DRB1*11	0,178	0,242
DRB1*12	0,015	0,015
DRB1*13	0,232	0,203
DRB1*14	0,178	0,132
DRB1*15	0,155	0,171
DRB1*16	0,124	0,171
HLA-DQ1*		
DQB1*02	0,31	0,234
DQB1*03	0,736	0,867
DQB1*04	0,217	0,21
DQB1*05	0,41	0,281
DQB1*06	0,325	0,406
